# A novel accessory gene product of tick-borne Dhori-Orthomyxovirus, encoded by overlooked spliced transcripts of RNA segment 6

**DOI:** 10.1128/jvi.00600-25

**Published:** 2025-09-15

**Authors:** E. Bendl, G. Lampo, P. Chlanda, E. Schnettler, G. Kochs, J. Dengjel, L. Graf

**Affiliations:** 1 Institute of Virology, Freiburg University Medical Center, Faculty of Medicine, University of Freiburg 9174, Freiburg, Germany; 2 Department of Biology, University of Fribourg 9174, Fribourg, Switzerland; 3 Department of Infectious Diseases-Virology, Medical Faculty, Heidelberg University 9144, Heidelberg, Germany; 4 BioQuant, Heidelberg University 9144, Heidelberg, Germany; 5 Bernhard-Nocht- Institute for Tropical Medicine 14888, Hamburg, Germany; 6 Faculty of Mathematics, Informatics and Natural Sciences, University of Hamburg, Hamburg, Germany; 7 German Centre for Infection Research (DZIF), partner site Hamburg-Luebeck-Borstel-Riems 459706, Hamburg, Germany; University of North Carolina at Chapel Hill, Chapel Hill, North Carolina, USA

**Keywords:** orthomyxoviruses, thogotoviruses, Dhori virus, Bourbon virus, tick-borne, arbovirus, matrix protein, new accessory viral gene product

## Abstract

Dhori virus (DHOV) (*Orthomyxoviridae,* genus: Thogotovirus) is a tick-borne virus with a segmented, negative-sense RNA genome. Thogotoviruses can be divided into two clades, Thogoto-like and Dhori-like viruses, which differ in their coding strategy of the segment 6 encoded matrix protein (M): Thogoto viruses translate M from spliced transcripts and a long M (ML) from unspliced transcripts. Dhori-like viruses encode their M using unspliced transcripts, whereas no splicing or additional coding capacity has been described, yet. Here, we identified spliced transcripts of segment 6 in DHOV-infected cells by RT-PCR and sequencing. The gene product M2-248 codes for a truncated, 186 amino acids-long N-terminal moiety of M fused to 62 C-terminal amino acids from a −1 shifted reading frame. The splicing sites and the amino acid sequences of the C-terminal M2 part are conserved in Dhori-like viruses. Expression of M2-248 was confirmed in infected cells by mass spectrometry and western blot analysis and was detected in purified virions. Recombinant DHOV(ΔM2) lacking M2-248 showed reduced virulence compared with recombinant wild-type in mice but no attenuation in cell culture and no effect on virion assembly and morphology. An interferon antagonistic function of M2-248, similar to THOV ML, was excluded. Thus, our data suggest that DHOV segment 6 splicing and expression of M2-248 protein affect virulence in mammals by a mechanism distinct from THOV-ML. It remains to be analyzed how M2-248 might affect viral host switch and replication in ticks.

**IMPORTANCE:**

Dhori and Thogoto viruses are tick-transmitted orthomyxoviruses comprising two clades differing in the coding strategy of their matrix protein. The recent detection of Dhori-like Bourbon virus (BRBV) highlighted their zoonotic potential. M of Dhori-like viruses is translated from a collinear transcript of the smallest of the six genomic ssRNA segments. Thogoto-like viruses express M from spliced transcripts, and unspliced transcripts encode an extended ML protein. No analogous splicing event or additional gene product has been characterized for Dhori-like viruses, yet. Here, we describe the hitherto overlooked splicing of segment 6 transcripts of Dhori virus. The 5'-part of the processed transcript is collinear with the M-ORF, whereas splicing shifts it into a -1 frame. The predicted product M2-248 was detected in infected cells. Recombinant Dhori virus lacking M2 was attenuated *in vivo*, whereas replication in mammalian cells was not impaired, suggesting a modulatory function of M2 in *in vivo*-specific, cellular immunity-related processes.

## INTRODUCTION

Thogotoviruses with their segmented, negative sense-ssRNA genome form a separate genus within the family of *Orthomyxoviridae*. Each of the six segments codes for one structural protein (1): the three subunits of the viral polymerase, PB2, PB1, PA, the viral glycoprotein (GP) inserted into the viral envelope, the nucleoprotein (NP), and the matrix protein (M). A characteristic feature of thogotoviruses is their transmission by ticks and their capacity to replicate in ticks as well as in mammals (2–4). Accordingly, the viral glycoprotein (GP) structurally resembles that of insect baculoviruses (5), enabling thogotoviruses to infect tick as well as mammalian cells. During the last years, several thogotoviruses were isolated, mostly from infected ticks collected in different parts of the world, and serological studies showed thogotovirus infections in various wild and domestic mammalian species including humans (1). Phylogenetic and serological analyses led to the classification of thogotoviruses into two clades (1, 6): The Dhori-like and the Thogoto-like viruses, with the prototype species DHOV/India/1313/61 (7) and THOV/SiAr/126/72 (8).

Historic reports described five laboratory human infections with DHOV in the former Soviet Union provoking febrile illness and encephalitis (9). More recently, several tick-borne zoonotic infections with the Dhori-like Bourbon virus (BRBV) were reported in the United States with occasionally severe outcomes (10–12). The pathogenesis of DHOV was experimentally studied in mice. Intraperitoneal or subcutaneous infections of laboratory animals led to severe clinical outcomes with high viral loads in the spleen, lungs, and liver, exceeding cytokine production and death from lung and liver damage within 4–5 days (6, 13–15).

**Fig 1:**
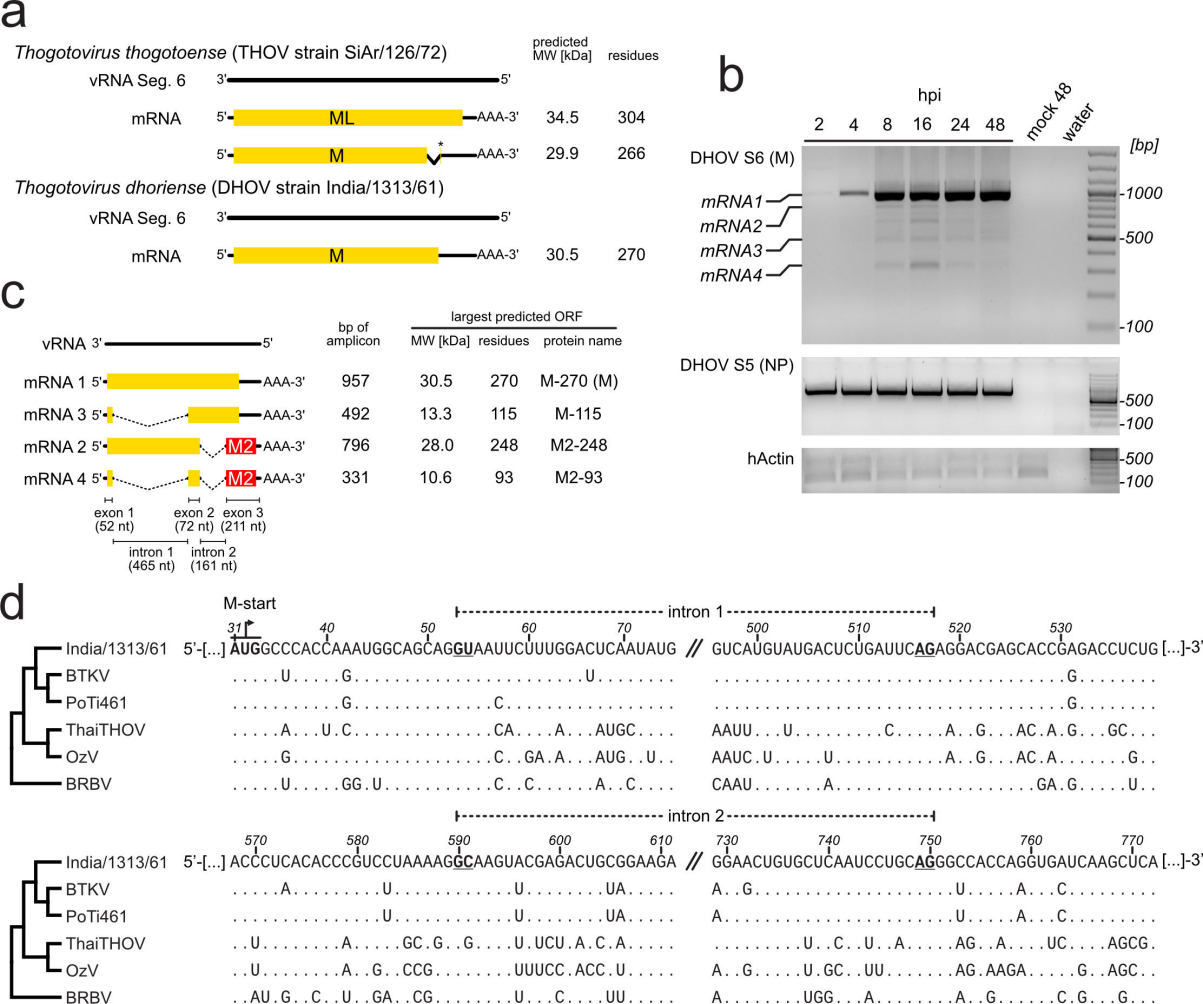
DHOV segment 6 transcripts are spliced in infected cells. (a) Schematic of canonical THOV and DHOV segment 6 coding strategy. The star (*) indicates the formation of a stop codon by the splicing event. The coding capacity of the ORFs (yellow) and the predicted molecular weights are indicated for the two prototype strains of Thogoto- and Dhori-like viruses. (b) RT-PCR analysis of segment 6 (S6) transcripts from DHOV-infected cells. Huh7 cells were infected with India/1313/61 at a moi of 0.5 for 1 h. The inoculum was removed, and the cells were washed and incubated with fresh medium at 37°C. Poly A-positive RNA was isolated from the cells at the indicated time points post-infection (hpi). Unspliced (full length) transcripts coding for the matrix protein (M) were expected to yield amplicons of 957 bp (mRNA 1). Additional PCR products representing putative splice products (mRNA2–4) were obtained. Segment 5 (S5) coding for NP and β-actin-specific primer sets were included as controls. The sizes of DNA marker fragments are indicated. The panel shows the representative result of two independent experiments. (c) Schematic of the mRNA transcripts of segment 6 from DHOV-infected cells identified by RT-PCR and Sanger sequencing: mRNA 1, unspliced, with the matrix protein (M) reading frame (yellow); mRNA 3, splicing of the first intron; mRNA 2, splicing of the second intron leading to −1 reading frame M2 (red); and mRNA 4, splicing of both introns. Introns are indicated by dotted lines. The sizes of exons and introns are indicated below; the amplicon sizes and the predicted lengths and molecular weights of the translation products are on the right. (d) cRNA sequence alignment indicates conserved splicing sites in segment 6 of Dhori-like viruses. Segment 6 cRNA sequences of DHOV strains India/1313/61 [MT628433.1], BTKV [MT628421.1], and PoTi461 [MT628427.1], Thailand thogotovirus isolate [MN095544.1], OzV [LC320128.1], and BRBV [KP657750.3]. The AUG start codon of the M-ORF and the splice donor and acceptor sites (first and last two nucleotides for each intron) are highlighted. Identical nucleotides as in the India/1313/61 sequence are indicated by dots. The left-hand cladogram indicates the phylogenetic relationship between the Dhori-like isolates. The numbers refer to the positions of the genomic segment 6 vRNA of DHOV/India/1313/61.

Members of the *Orthomyxoviridae* expand their coding capacity by utilizing host splicing machinery to produce multiple transcripts generated from certain genome segments. This process has been well described for the influenza A virus (IAV) M and NS segments (16). Accordingly, it has been shown that the M protein of Thogoto-like viruses is translated from a spliced transcript of segment 6, and the splicing event creates a stop codon to terminate the M open reading frame (ORF) (Fig. 1a). The unspliced transcripts of THOV segment 6 encode the M-ORF C-terminally elongated by 38 amino acids, called ML (17, 18). The ML gene product, and especially its C-terminal extension, acts as a viral interferon (IFN) antagonist (17, 19). However, previous studies established that Dhori-like viruses express their M from an unspliced transcript of segment 6 (20, 21) and that DHOV segment 6 does not undergo splicing. Segment 6 of DHOV contains 961 nucleotides (nt) and codes for an M protein of 270 amino acids, called M-270. In DHOV/India/1313/61 and Oz virus (OzV)-infected cells, only a single transcript species corresponding in size to the genomic RNA of segment 6 was previously described (20, 21). However, in their characterization of the DHOV matrix protein, Clay and Fuller described subgenomic transcripts of segment 6 in low abundance detected in overexposed Northern blot analyses and proposed a second ORF in segment 6. This 327 nucleotides-long ORF in a −1 frame partly overlapped with the M-ORF and had a coding capacity of 141 amino acids. The expression of this putative second gene product could not be verified (20).

This prompted us to reevaluate the coding capacity of DHOV segment 6 by analyzing viral transcripts from DHOV/India/1313/61 infected cells. Interestingly, we detected mRNA processing by two independent splicing events. The distal splicing resulted in a −1-frame shift compared with the M-ORF. Accordingly, the removal of the second intron truncated the M-ORF to its N-terminal 186 amino acids before shifting into the −1 frame that elongated the polypeptide by a unique C-terminal extension of 62 amino acids. This second gene product of segment 6, referred to as M2-248 from here on, is dispensable for viral replication and lacks IFN-antagonistic activity *in vitro*. However, recombinant DHOV(ΔM2) lacking M2-248 expression showed an attenuated phenotype in mice, indicating that M2 is a significant virulence factor.

## RESULTS

### Detection of subgenomic transcripts of segment 6 in DHOV-infected cells

We re-evaluated the transcripts of segment 6 isolated from DHOV-infected human hepatoma Huh7 cells by RT-PCR using random hexamers and an oligo dT primer mix for cDNA synthesis and a segment 6 specific primer pair amplifying the near full-length segment 6 in an amplicon of 957 nt. The agarose gel analysis of the PCR products showed the expected prominent band corresponding to the full-length transcript (mRNA1), but some additional smaller bands (mRNA2-4) down to about 300 nt in length (Fig. 1b), indicating spliced products of mRNA1. Visible bands were cut out from the gel and subjected to Sanger sequencing, which returned four cDNAs showing similarities to DHOV segment 6 predominantly in the 5´- and 3´-end sequences (Fig. 1c). Additional bands visible in the agarose gel returned inconclusive sequencing data and might represent splicing intermediates or unspecific amplification of other viral RNAs due to the high similarity of the 3’- and 5’-non-coding regions to which the primers partly bind.

mRNA1 was verified as the full-length transcript of segment 6, and the sequences of mRNA2, 3, and 4 partially overlapped with mRNA1 but were reduced in length by two distinct deletions: mRNA3 and mRNA4 showed a 465 nt deletion from nts 53 to 517, and mRNA2 and mRNA4 a 161 nt deletion from nts 590 to 750 (Fig. 1c and d). Accordingly, the predicted ORF of mRNA1 codes for the 270 amino acids (aa) of the matrix protein (from here on also referred to as M-270) and mRNA3 for a truncated version of M lacking 155 aa (M-115). Removal of the second intron resulted in a −1 shift of the M-ORF into the new, 189 nt-long M2 reading frame coding for 63 codons (Fig. 1c). Single spliced mRNA2 codes for a truncated M protein of 186 N-terminal aa continued by 62 M2-specific aa (hereafter M2-248). The double-spliced mRNA4 codes for a predicted peptide of 31 N-terminal aa of the M-ORF continued by the 62 M2-specific aa (M2-93).

An alignment of the segment 6 nucleotide sequences publicly available for Dhori-like viruses showed an overall high similarity of >63%, including a nearly complete conservation around the two putative splice donor and acceptor sites (Fig. 1d).

To investigate whether splicing of DHOV segment 6 also occurs not only in mammalian but also in tick cells, we chose two tick cell lines from *Hyalomma anatolicum* (HAE/CTVM9) and *Rhipicephalus appendiculatus* (RAE/CTVM1) because both Ixodid tick genera had been described to host Dhori-like viruses (22, 23; reviewed in reference 1). We infected both cell cultures with DHOV/1313/16 at a moi of 0.1 and harvested RNA from the infected cells at 8 dpi. A conventional RT-PCR analysis targeting segment 5 demonstrated the presence of viral RNA in the cells (Fig. S1a). A segment 6 spanning RT-PCR (as in Fig. 1b) demonstrated the accumulation of all expected transcripts of segment 6 comparable with the transcripts in Huh7 cells infected for 16 h: unspliced mRNA1, intron 1-spliced mRNA3, intron 2 spliced mRNA2, and double spliced mRNA4. Finally, the splicing of segment 6 transcripts in the infected HAE cells was confirmed by next-generation deep sequencing of the viral RNAs (Fig. S1b).

### Detection of M2 protein expression in DHOV-infected cells

**Fig 2:**
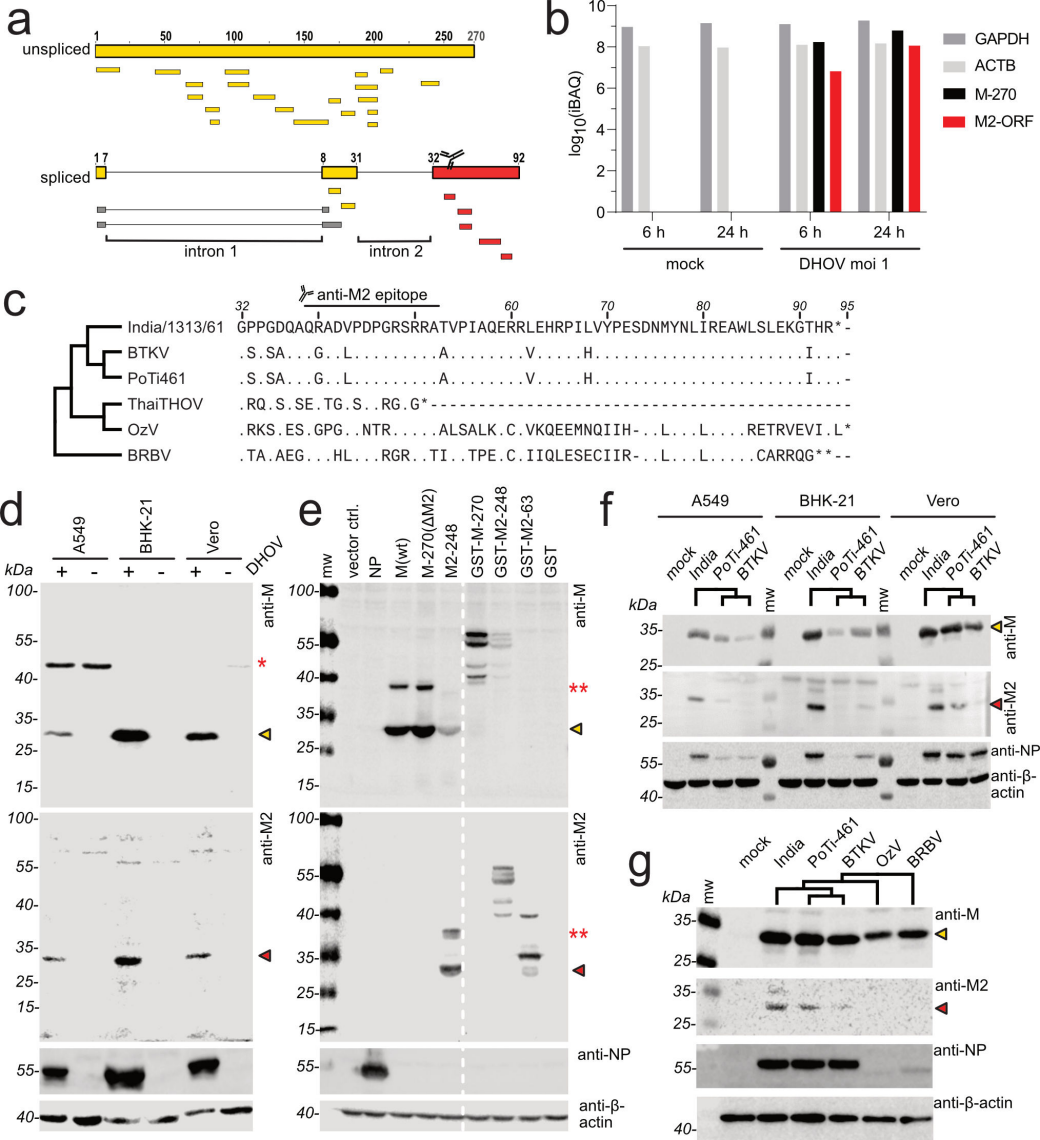
Identification of an M2 gene product in DHOV-infected cells. (a) Schematic of the M-270 and the putative M2-93 translation products from the unspliced and double-spliced transcripts of segment 6. The bold upper bars correspond to the M-270-ORF (yellow), and the double-spliced mRNA4 translation product with the unique C-terminal M2 part (red). The intronic regions are indicated by black lines. The smaller bars below indicate the peptides detected by the MS/MS analysis, corresponding to the reading frames of the canonical M-ORF of DHOV (yellow), the intron 1 spanning region (gray), and the M2 part (red). The numbers indicate the amino acid positions in the ORFs. The antibody symbol indicates the position of the antigenic peptide recognized by the M2-specific antibody. (b) Peptide-based protein abundances inferred from the MS/MS analysis of A549 cells mock-infected or infected with DHOV (moi 1) for 6 and 24 h. IBAQ (intensity-based absolute quantification) values as an approximate of protein abundance of viral M-270 and the M2 part were compared with those of cellular GAPDH and beta-actin (ACTB). (c) Amino acid alignment of the unique M2 region of Dhori-like viruses. The sequences are aligned to the DHOV/India/1313/61 reference sequence (GenBank: MT628433.1) and the numeration corresponds to the amino acid position in the deduced, M2-93-ORF encoded by the double-spliced transcripts. Conserved residues are indicated by dots, missing residues by hyphens, and stars indicate termination of the ORF by a stop codon. For segment 6 of ThaiTHOV, only a truncated cDNA sequence is available (GenBank: MN095544.1), and the M2-ORF is terminated by a premature stop codon, and therefore, its M2 region is shortened by 43 amino acids compared with the India/1313/61 M2-93. (d) Detection of M-270 and M2-248 by western blot analysis of lysates of mammalian A549, BHK-21, and Vero cells either mock-treated (-) or infected with DHOV/India/1313/61 (+) with moi 1 for 24 h. (e) Expression of recombinant M-270 and M2-248. 293T cells were transfected for 48 h with expression constructs coding for DHOV-NP, DHOV-M cDNA (M(wt)), M-270(ΔM2) cDNA with inactivating mutations in the splice acceptor site of intron 1 and the splice donor and acceptor sites of intron 2, and for the M2-248 coding cDNA with an inactivating mutation in the splice acceptor site of intron 1. Furthermore, expression constructs encoding GST-fusion constructs of M-270, M2-248, and M2-63 were transfected, the latter containing only the C-terminal 62 amino acids of the M2-specific region fused to GST. (panels d and e) The western blots were incubated with polyclonal rabbit antisera raised against the matrix protein purified from extracellular DHOV/India/1313/61 virions (anti-M, yellow arrowheads) or against a synthetic M2 peptide (see panel 2 c, anti-M2, red arrowheads). The red asterisk in panel (d) marks an upper band of about 45 kDa observed also in uninfected A549 cells. Two red asterisks in panel (e) mark additional upper bands of M and M2 in transfected cells. A viral NP-specific antiserum and a β-actin antibody were used as an infection control and as a loading control, respectively. (f) Western blot analysis of mammalian cells infected with DHOV strains: India/1313/61 (India), PoTi-461, and Batken virus (BTKV). Cells were infected with moi 1 and lysed 24 hpi. (g) Western blot analysis of Vero cells infected and processed as in panel f with strains of DHOV as well as Dhori-like OzV and BRBV. mw = molecular wt marker.

To get the first evidence for the expression of the splice products containing the M2-specific region, we analyzed lysates of DHOV-infected human lung epithelial A549 cells by mass spectrometry (MS). The MS/MS analysis detected peptides spanning the entire M-270 protein but also peptides corresponding to the unique, C-terminal part of the M2-specific region (Fig. 2a, yellow and red small bars, respectively). Intriguingly, the MS analysis also detected two peptides that consist of amino acid sequences from exon 1 and exon 2 (gray small bars), suggesting the translation of spliced intron 1 transcripts. We quantified the relative levels of peptides derived from host and viral proteins based on iBAQ (intensity-based absolute quantification) values, confirming an abundant presence of viral matrix protein (M-270) as well as M2-containing viral gene products in infected cells (Fig. 2b).

Subsequently, we intended to prove M2 expression in infected cells using western blot analysis. Thus, polyclonal rabbit antisera were raised against the matrix protein purified from extracellular DHOV/India/1313/61 particles (anti-M) or against a 14 aa-long synthetic peptide, corresponding to the unique, C-terminal part of M2 (Fig. 2c, anti-M2 epitope). Different mammalian cell lines were infected with DHOV/India/1313/61, and the cell lysates were analyzed using gel electrophoresis and western blot. The polyclonal anti-M antiserum recognized a protein of about 29 kDa in the lysates of DHOV-infected, but not of mock-treated, cells (Fig. 2d). This protein was also observed in the lysates of 293T cells transfected with M-270 and M2-248 encoding expression plasmids (Fig. 2e). The size of about 29 kDa corresponds to the predicted molecular weight of M-270 and coincides with the molecular weight determined previously for purified DHOV M (20). Higher bands of about ~10 kDa above the predicted molecular weights of M and M2 were especially observed in transfected cells (Fig. 2e, two red asterisks). We speculate that the upper band of about 39 kDa corresponds to post-translationally modified versions of the M or M2 protein. Furthermore, the polyclonal M-specific antiserum recognized M2-248 (Fig. 2e), most likely by binding to epitopes localized in its N-terminal part that corresponds to the N-terminus of M-270 (see scheme in Fig. 1c). Accordingly, the M2-specific 63 aa of a GST fusion protein (GST-M2-63) was not recognized by the anti-M antiserum (Fig. 2e). Although the MS/MS analysis confirmed the presence of peptides derived from intron 1-spliced transcripts, we did not detect protein bands corresponding to the predicted molecular weights of M-115 or M2-93 in western blot analyses (Fig. 2d).

The M2-specific antiserum recognized M2-248 but not M-270 (Fig. 2d, lower panel). Again, the western blot analysis (Fig. 2e, lower panel) of transfected cells showed two bands of about 28 and 38 kDa, corresponding to the predicted molecular weight of M2-248 and its possibly modified version (Fig. 2e, two red asterisks). Additionally, the two antibodies recognized M-270 and M2-248 in lysates of different mammalian cells infected with three different isolates of DHOV (Fig. 2f and g). However, an M2-248 protein was not detected in the lysates of Dhori-like OzV and BRBV-infected cells, although the expression of M-270 could be detected by the anti-M serum. The dissimilarity of the M2 amino acid sequences of the two Dhori-like viruses when compared with the DHOV sequences in the antigenic M2 peptide region (Fig. 2c) most likely accounts for the lack of M2-specific antibody recognition. This antigenic heterogeneity between DHOV and Dhori-like viruses was also evident for the viral NP protein (Fig. 2g).

### Structure prediction and analysis of M2-248

AlphaFold 3 was used (24) to predict the structures of DHOV M-270 and M2-248 as well as THOV M and ML (Fig. S2a). Overall, the predicted DHOV M-270 as well as the THOV M structures show a two-domain architecture similar to the M1 protein of influenza A virus (25, 26). The N-terminal domains (NTD) and a more variable fold of the C-terminal domain (CTD) were predicted to be formed by alpha-helices linked by a disordered region. It is noteworthy that the predicted structure of the N-terminal domain of THOV M matches the structure determined using X-ray crystallography (PDB: 5I5N) (27). Prediction confidence of the NTD (aa 13–140) of DHOV M-270 and M2-248 and CTD of M-270 (aa 148–270) was high, whereas low prediction confidence was reported for the disordered “linker” region (aa 141–167) (Fig. S2b). However, the C-terminal domain of M2-248 (aa 148–248) was predicted with only medium-to-low confidence that increases for the predicted C-terminal α-helix (Fig. S2b). A hydrophobicity plot (Fig. S2c) of DHOV M-270 and M2-248 predicts similar biophysical properties for the N-terminal part and the linker region with a single hydrophobic stretch (aa 115–141) already described by Clay and Fuller (20). Interestingly, the C-terminal regions showed different profiles (Fig. S2c). None of the two proteins contained predicted transmembrane structures according to the DeepTMHMM v1.0 (28) prediction tool (not shown). However, both M-270 and M2-248 contain a predicted amphipathic helix at their C-termini (Fig. S2d, in gray) in addition to two putative late domain motifs (YQIL and YQLL) in M-270. These late domain motifs are conserved in the M protein sequences of all DHOV and Dhori-like sequences (not shown) and are commonly associated with the budding and release of enveloped RNA viruses (29).

In summary, our molecular analyses of DHOV-infected cells confirmed the synthesis of a new viral gene product, called M2-248, that consists of the N-terminal part of the matrix protein fused to a unique M2 structure translated from a shifted reading frame caused by a splicing event.

### Generation of a recombinant DHOV lacking M2 expression (ΔM2)

To evaluate the effect of the new gene product, M2-248, on DHOV replication, we established a reverse genetic system to rescue recombinant rDHOV. For this, the six segments of DHOV strain India/1313/61 were amplified by RT-PCR, and the cDNAs are cloned into the pHW2000 ambisense vector (30). Generation of rDHOV was performed as previously described for rTHOV (19). Virions harvested from the transfected cell cultures were plaque purified, and virus stocks were prepared on BHK-21 cells.

**Fig 3:**
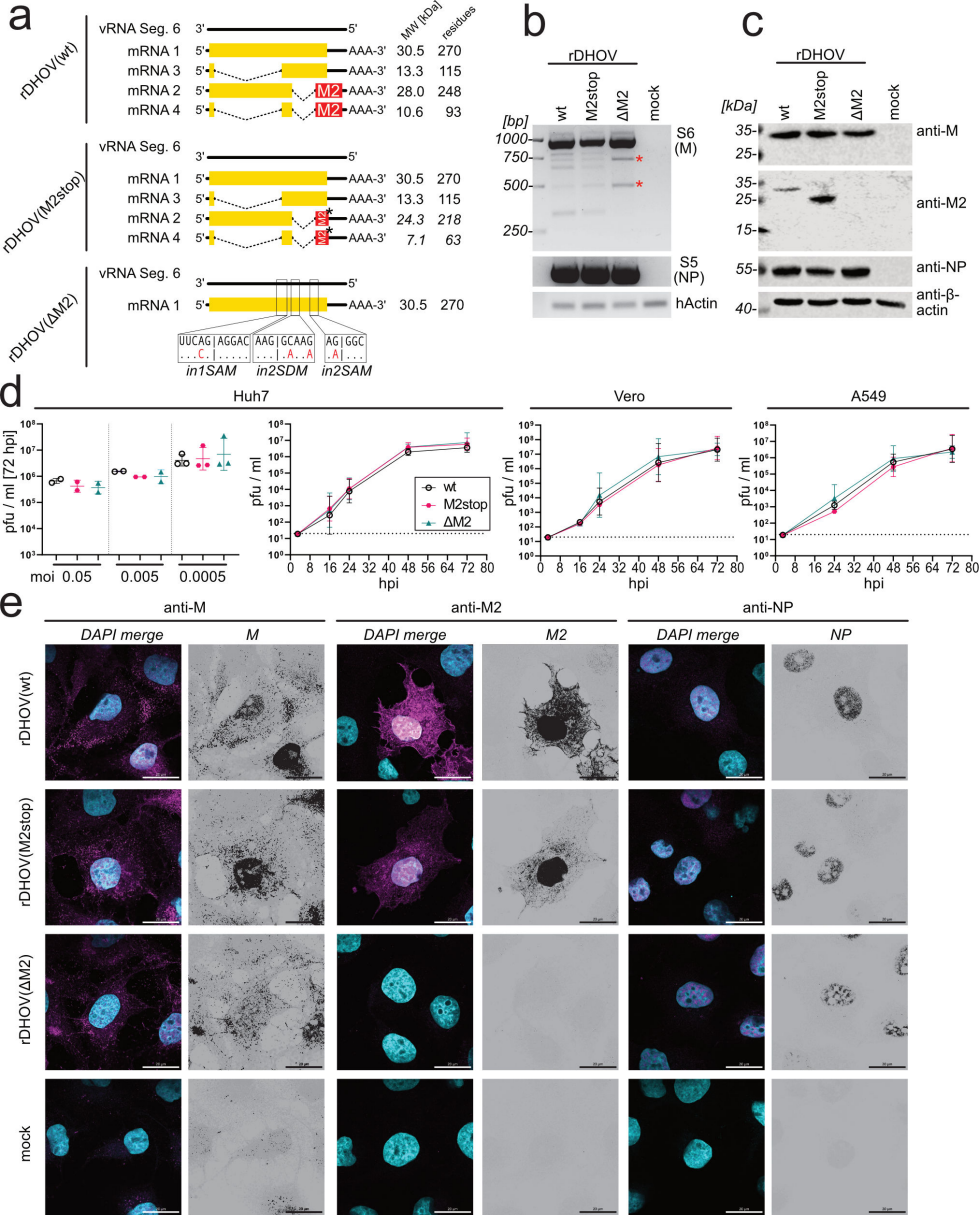
Generation and characterization of recombinant M2-deficient viruses. (a) Schematic of the genomic vRNA and the expected mRNA transcripts of recombinant DHOV (rDHOV). rDHOV(wt) encodes the unaltered vRNA of DHOV/India/1313/61 segment 6. rDHOV(M2stop) encodes a premature stop codon (star) at position 219 of the M2-ORF, leading to a truncation of M2 by 30 amino acids resulting in M2-218. To generate rDHOV(ΔM2), the splice acceptor site of intron 1 (in1SAM) and the splice donor (in2SDM) and acceptor sites (in2SAM) of intron 2 were inactivated as indicated below. Predicted molecular weights and amino acid residues are indicated on the right. (b) RT-PCR analysis of segment 6 transcripts from rDHOV-infected Huh7 cells. Cells were infected with a moi of 0.5 for 24 h. Full-length segment 6 transcripts were analyzed by RT-PCR as described in Fig. 1b. The red stars indicate two amplicons corresponding to unconventional intron one splice products. Segment 5 (S5) and β-actin-specific primer sets were included as controls. (c) Western blot analysis of rDHOV-infected Huh7 cells. Cells were infected with a moi of 0.1, and whole cell lysates were obtained at 36 hpi. Viral proteins were detected using M-, M2-, and NP-specific antisera. Detection of β-actin was used as a loading control. (d) Growth kinetics of rDHOV in mammalian cell lines. Huh7 cells were infected with different MOIs for 2 h. Afterward, the cells were washed with PBS, and the inoculum was replaced by a fresh medium. Progeny virus in the supernatants was quantified by plaque assay at 72 hpi. Accordingly, Huh7, A549, and Vero cells were infected with a moi of 0.0005, and progeny virus production was determined at the indicated time points. Error bars represent the SD of three independent experiments. Each experiment included technical duplicates. The dotted lines indicate the detection limit. (e) Immunofluorescence analysis of Huh7 cells infected with moi 2 of rDHOV(wt), rDHOV(M2stop), or rDHOV(ΔM2) or mock-treated. At 24 hpi, the cells were fixed with paraformaldehyde and stained with antibod**i**es specific for viral M, M2, and NP. Nuclei were stained for genomic DNA using DAPI; 63 × magnification; and scale bars indicate 20 µm. The panel shows representative images from three independent experiments.

The segment 6 coding capacity and design of three generated recombinant DHOVs is summarized in Fig. 3a. Segment 6 of rDHOV(wt) corresponds to the sequence of the parental DHOV/India/1313/61 encoding the entire M and the various M2 gene products by functional splice sites of intron 1 and intron 2. Segment 6 of rDHOV(M2stop) also encodes functional intron 1 and intron 2 splice sites, but a truncated M2-ORF by introducing a premature stop codon that terminates the M2-ORF 30 codons before its authentic stop codon, resulting in a shortened, 218 aa-long M2-218 protein, without affecting the sequence of the M protein. Segment 6 of rDHOV(ΔM2) lacks the expression of spliced mRNAs and, therefore, the expression of the protein products M2-93 and M2-248, containing the M2 region, by mutating the splice acceptor site of intron 1 as well as the splice donor and splice acceptor sites of intron 2. The splice donor site of intron 1 could not be incapacitated without resulting in amino acid changes affecting the M-ORF. To verify the expected coding capacities of the three recombinant viruses, Huh7 cells were infected with the recombinant viruses, and the expression patterns of segment 6 were analyzed using RT-PCR of the viral transcripts (Fig. 3b) and using western blot to detect the viral proteins (Fig. 3c). The gel electrophoresis pattern of the smaller, spliced transcripts appeared identical for rDHOV(wt) and rDHOV(M2stop). In contrast, rDHOV(ΔM2) lacked amplicons of intron 2 spliced transcripts (Fig. 3b). However, in rDHOV(ΔM2)-infected cells, we detected two cDNA amplicons of about 500 and 800 bp (Fig. 3b, red asterisks). Sanger sequencing of these cDNAs confirmed the lack of intron 2 splicing but indicated sporadic splice events in intron 1. This suggests that inactivation of the intron 1 splice acceptor site did not totally suppress splicing and instead led to the use of alternative splice-acceptor sites. As a control, amplicons of segment 5 of about 1,500 nt were amplified from the isolated RNAs (lower panel), confirming comparable infection efficiency. The western blot analyses of viral proteins accumulated in infected cells 24 h post-infection showed equal production of M-270 and NP proteins (Fig. 3c). However, the M2-248 band detected in the rDHOV(wt)-infected cells shifted from about 29 kDa to a reduced molecular weight of about 25 kDa in rDHOV(M2stop) infected cells and was absent in the rDHOV(ΔM2)-infected cell lysates (Fig. 3c), confirming the expected M2 expression patterns of the recombinant viruses.

Next, we analyzed the capacity of the three recombinant viruses to replicate in mammalian cell cultures. Infection of the cells with a low moi of 0.0005 for 72 h led to progeny release of about 6 × 10^7^ pfu/mL for all three rDHOVs (Fig. 3d). Infection with this low moi revealed growth kinetics comparable for all three rDHOV over 72 h in Huh7, Vero, and A549 cells (Fig. 3d). To examine the intracellular localization of the viral proteins, Huh7 cells were infected with the rDHOVs at a higher moi of 2 that resulted in almost complete infection efficiency. Immunofluorescence microscopy of the fixed cell cultures at 24 hpi using specific antibodies showed nuclear staining of NP as well as M protein staining in the nuclear and the cytoplasmic compartments in almost all cells (Fig. 3e). Interestingly, the staining with the M2-specific antiserum showed a lower number of positive cells with some strong signals in the nucleus and a distinct web-like, cytoplasmic localization for the wild-type as well as for the truncated M2stop. Both M and M2 were also detected at the plasma membrane of the infected cells. Overall, our comparative analysis of rDHOV(ΔM2) with rDHOV(wt) suggests that M2-248 expression does not grossly affect viral replication in mammalian cell cultures.

### M2-248 interacts with M and is incorporated into released virions

**Fig 4:**
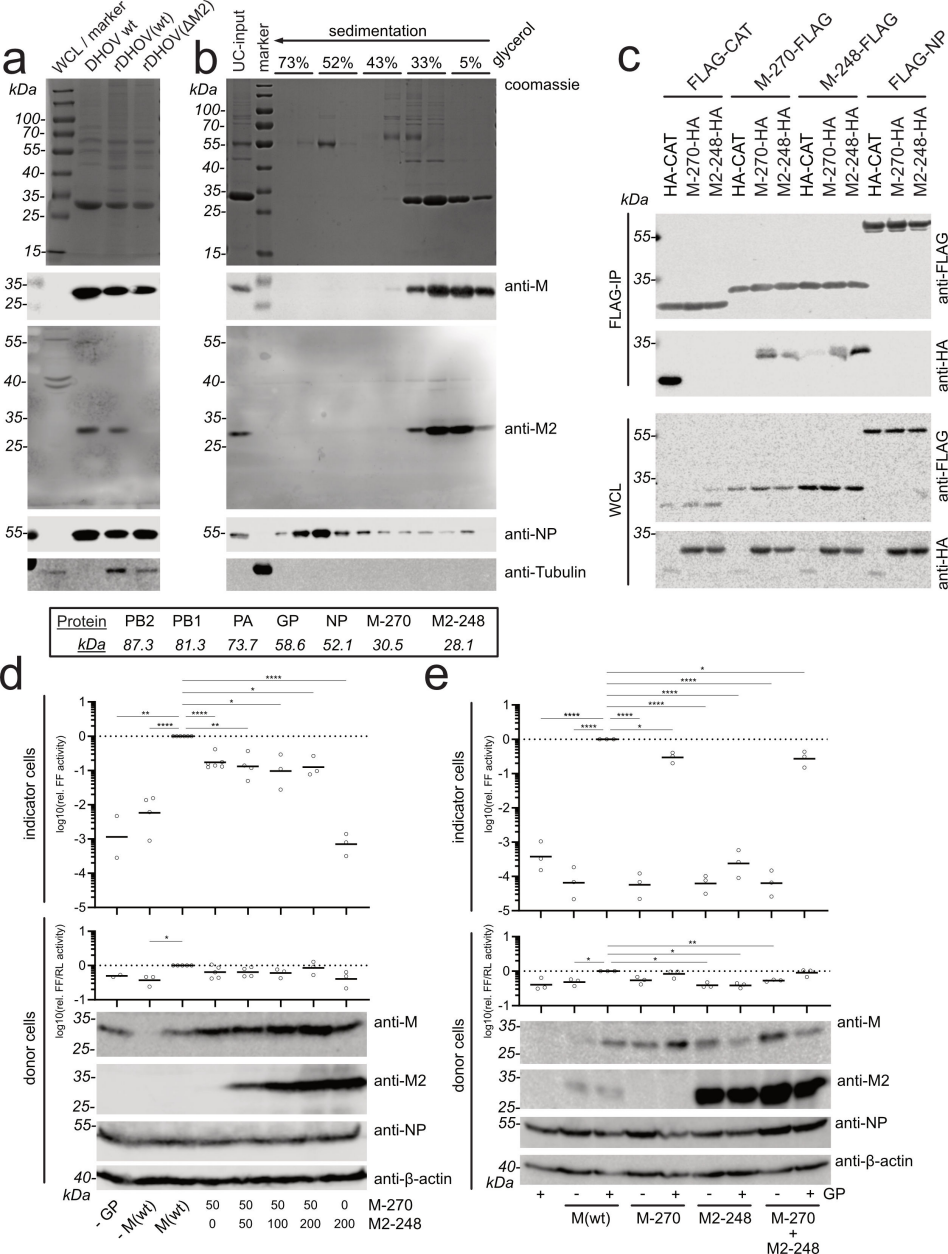
DHOV-M2-248 is a structural protein but is not required for particle formation. (a) Extracellular viral particles were harvested from the supernatants of DHOV/India/1313/61-infected BHK-21 cells and rDHOV(wt)- or rDHOV(ΔM2)-infected Huh7 cells. The purification procedure consisted of a three-step ultracentrifugation protocol, including first collection of the virions through a 30% glycerol cushion, separation of the virions through a 30%–60% sucrose gradient, and then collecting the virion fraction in a small volume by centrifugation through a 30% glycerol cushion. The purified fractions were resolved by SDS-PAGE and analyzed by Coomassie staining and western blot using M-, M2-, NP-, and β-tubulin-specific antibodies. Molecular weight markers are indicated on the left, and the predicted molecular weights of DHOV structural proteins are listed below. Whole cell lysates (WCL) of uninfected BHK cells were mixed in the marker lane. (b) The purified DHOV/India/1313/61 particles (3 × 10^7^ pfu) were lysed with detergent and subjected to ultracentrifugation through a 33%–73% discontinuous glycerol gradient. The gradient was fractionated into 10 fractions that were analyzed using SDS-PAGE, Coomassie staining, and western blot as described in panel a. (c) M2-248 interacts with M-270. 293T cells (~5 × 10^5^ cells/6-well) were transfected with 1 µg of each expression plasmid encoding C-terminally FLAG- or HA-tagged M-270 and M2-248. After 48 h, FLAG-tagged bait proteins were immunoprecipitated with anti-FLAG-IgG agarose beads. Precipitated FLAG-tagged bait or co-precipitated HA-tagged prey was analyzed using SDS-PAGE and western blot using FLAG- and HA-specific antibodies. The blots show a representative result out of three individual experiments. CAT, chloramphenicol acetyltransferase. (d, e) Formation of DHOV-VLPs. 293T cells were transfected with pCAGGS expression plasmids encoding the structural proteins of DHOV: PB1 (20 ng), PB2 (20 ng), PA (20 ng), NP (100 ng), GP (50 ng), as well as the gene products from DHOV segment 6: wildtype segment 6 encoding M-270 and M2-248 in a splicing-dependent manner (M(wt), 50 ng), M-270 with inactivated splice sites of intron 1 and 2 (50 ng), or M2-248 with an inactivated intron 1 splice site (100 ng). In panel d, various amounts of the M2-248 encoding plasmid were used. In panel e, VLP formation with or without GP was examined. A viral polymerase-dependent reporter plasmid encoding firefly luciferase (FF) gene flanked by the 5’- and 3′-NTRs from DHOV segment 5 (pPolI-FF, 75 ng) and a plasmid constitutively expressing *Renilla* luciferase (RL; SV40p-RL, 5 ng) as an internal control were co-transfected. Expression of the transfected cDNAs in the donor cells was monitored at 48 h post-transfection by western blot analysis using M-, M2- and NP-specific antibodies. Detection of β-actin served as a loading control. At 48 h post-transfection, supernatants of the transfected 293T cells were transferred to naïve BHK-21 indicator cells for 48 h. Donor and indicator cell lysates were checked for luciferase activities. FF luciferase activities of donor cells were normalized to RL luciferase activities and to the FF/RL values of M(wt)-transfected cells. FF luciferase activities of indicator cells were normalized to the activity of M(wt) VLPs in the indicator cells. Shown are the mean and individual data points for biological replicates with technical duplicates. Significance compared with M(wt) was calculated with a one-way ANOVA [Mixed effects model, *P* > 0.05 (ns), *P* ≤ 0.05 (*), *P* ≤ 0.01 (**), *P* ≤ 0.001 (***)].

Because of the structural similarity of M2-248 and M-270 (Fig. S2), we asked whether the former might be incorporated into viral particles as a structural protein. To investigate this, extracellular virus particles from the supernatants of DHOV/India/1313/61-infected BHK-21 cells were concentrated through a glycerol cushion and analyzed using western blot after a sucrose gradient fractionation. The analysis confirmed the presence of NP and M-270 as canonical structural proteins and, interestingly, also of the M2-248 protein as detected by the M2-specific antibody (Fig. 4a). As expected, extracellular virions of rDHOV(ΔM2)-infected cells lacked the M2-248 signal (Fig. 4a). As a control for contamination of our virion preparation with cellular components, we stained the western blot for cellular tubulin that was present in the mock-infected whole cell lysate (WCL) but not in the purified virions of the parental DHOV(wt). Because the virion preparations of rDHOV contained residual cellular debris as indicated by the detection of tubulin, the parental DHOV(wt) preparation that contained no detectable amounts of tubulin was used for the subsequent experiment.

For further analysis, the purified parental DHOV(wt) virions were lysed using detergent, and the structural components of the particles were separated by discontinuous 5%–73% glycerol density gradient centrifugation. Ten fractions were collected and analyzed for their protein content by SDS-PAGE and western blot. The analysis revealed sedimentation of NP (55 kDa) to the bottom of the gradient, most likely in the form of vRNA-associated protein forming dense vRNP structures. In the middle of the gradient, a signal around 68 kDa might reflect the sedimentation of the viral glycoprotein, and in the most upper fractions, around 34 kDa, the viral M-270 protein (Fig. 4b). Interestingly, the western blot demonstrated that M2-248 is also present in these M-270-positive fractions (Fig. 4b), suggesting that M2 is a structural viral component and might co-sediment in the glycerol gradient due to its interaction with the M protein. As mentioned before, we were not able to detect protein products of intron 1-spliced transcripts of segment 6 in this analysis.

A common feature of orthomyxoviral matrix proteins is their capacity to oligomerize, a requirement for the formation of the matrix layer beneath the envelope of orthomyxoviruses (25, 27). Therefore, we used co-immunoprecipitation to check whether M-248 can interact with M-270 in transfected cells. For this, the cDNAs coding for M-270 and M2-248 were C-terminally fused to the cDNA encoding a FLAG- or an HA-tag, respectively. Transfected 293T cells co-expressing these constructs were used for co-precipitation using anti-FLAG IgG-coated agarose beads. Accordingly, FLAG-tagged M-270 and M2-248 proteins were precipitated from the transfected cell lysates together with HA-tagged M-270 and M2-248 (Fig. 4c). As specificity controls, we used FLAG-tagged viral NP and chloramphenicol acetyltransferase (CAT) as well as HA-tagged CAT. These control proteins showed no precipitation of HA-tagged M-270 or M2-248, confirming a specific interaction between M-270 and M2-248.

The presence of M2-248 in extracellular particles implies that it might be involved in virion assembly and budding. To test this hypothesis, we established a system to reconstitute infectious virus-like particles (VLPs). For the expression of the recombinant proteins, the ORFs coding for the structural viral proteins, PA, PB1, PB2, GP, NP, and M(wt), were cloned into eukaryotic expression vectors. The M(wt) cDNA encodes the ORF of the authentic M gene with functional splice sites of intron 1 and 2, resulting in M-270 as well as M2-248 expression. Splicing of transcripts of the M-270 and M2-248-ORFs was prevented by the inactivation of the respective splice sites as mentioned above for the construction of the rDHOV rescue plasmids (Fig. 3a). In addition, the cDNA of an artificial genomic segment encoding firefly luciferase flanked by the non-coding regions of segment 5 was cloned into an RNA-polymerase I expression plasmid, resulting in the expression of a genomic vRNA segment of about 1,700 nt that can be transcribed by the reconstituted viral polymerase complex. Co-transfection of the complete set of viral structural proteins and the firefly luciferase vRNA into 293T donor cells resulted in the production of firefly luciferase, indicating the reconstitution of vRNPs with a functional viral polymerase complex (Fig. 4d, donor cells). Expression of the recombinant proteins in the transfected donor cells was confirmed by western blot analysis with M-, M2-, and NP-specific antibodies (Fig. 4d). The supernatants of these transfected donor cells were transferred to BHK-21 indicator cells to test for infectivity of the released VLPs by detecting the expression of firefly luciferase (Fig. 4d, upper panel). Omission of either the GP or the M(wt) expression plasmids did not grossly affect the expression of firefly luciferase in the donor cells but did not support the formation of VLPs and firefly luciferase activity in the indicator cells (Fig. 4d). Replacing M(wt) by the M-270 expression plasmid also supported VLP formation, resulting in firefly luciferase activity in the indicator cells, whereas M2-248 did not (Fig. 4d). Co-transfection of the M-270 plasmid with increasing amounts of M2-248-encoding plasmid had no effect on the reconstituted polymerase activity in the donor cells or on VLP formation detected in the indicator cells. The experiment depicted in Fig. 4e confirmed these results by showing the close dependency of VLP formation on GP and M-270 but not M2-248 expression. Likewise, attempts to rescue rDHOV with an M2-248 encoding bidirectional vector lacking intron 2, and therefore, M-270 expression were not successful (not shown).

### Structural analysis of budding and released DHOVs

**Fig 5:**
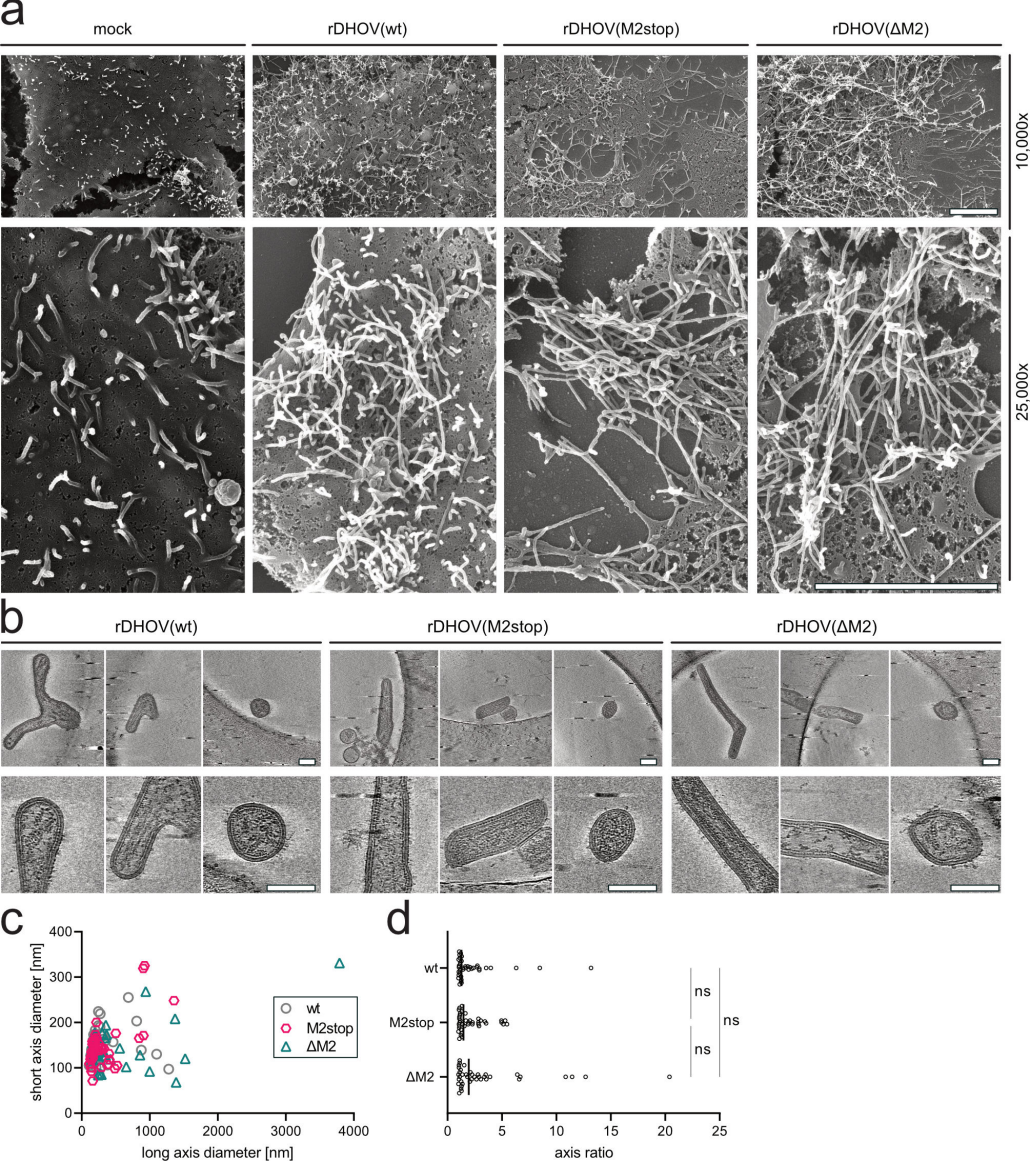
Scanning electron microscopy (SEM) and cryo-electron tomography (cryo-ET) analysis of DHOV particle morphology and the role of M2-248. (a) Budding phenotypes of rDHOV(wt), rDHOV(M2stop), and rDHOV(ΔM2) were analyzed by SEM of infected Huh7 cells seeded on indium tin oxide-coated coverslips. Cells were infected at moi ~4 or mock-infected as control and fixed at 24 hpi with 4% PFA and GA for 1 h at RT. Cells were imaged by SEM in an Aquilos cryo-FIB/SEM. Upper panel: 10,000× magnification, Lower panel: 25,000× magnification (scale bars 4 µm). Representative images are shown. (b) Cryo-ET of UV-inactivated rDHOV(wt), rDHOV(M2stop), and rDHOV(ΔM2) virions released into the supernatants. BHK-21 cells were infected with the respective viruses at moi 0.001 and incubated for 72 h in DMEM supplemented with 2% FCS, 20 mM HEPES, and 0.1% NaHCO_3_. Supernatants containing >1 × 10^6^ pfu/ml of infectious particles were UV-inactivated, plunge-frozen, and imaged by cryo-ET. (c) Quantification of viral particle shape from the samples in panel b as short axis vs. long axis diameters, and (d) the resulting axis ratios. Median and individual values (*n* = 40/virus) are displayed. Statistical analysis: Kruskal-Wallis Test and Dunn’s multiple comparisons test. ns, not significant.

To investigate whether M2-248, as a structural protein, might affect the budding phenotype and particle morphology, we performed scanning electron microscopy (SEM) and cryo-electron tomography (cryo-ET). Huh7 cells were infected with recombinant rDHOV(wt), rDHOV(M2stop), and rDHOV(ΔM2) for 24 h, fixed, and subjected to SEM sample preparation. The SEM images from the infected cells showed elongated budding profiles for all three viruses, indicating that M2 expression does not alter the budding process (Fig. 5a). The budding profiles can be distinguished from other physiological protrusions also observed in mock-treated controls. Those have a wider diameter and are shorter than budding virions and likely represent filopodia and microvilli (Fig. 5a). Furthermore, supernatants of infected BHK-21 cells were harvested and analyzed by cryo-ET. As reported previously for parental DHOV (6), the recombinant rDHOV(wt) forms spherical (120 nm in diameter) and long filamentous virions of up to 1,300 nm in length (Fig. 5b and c). The viral envelope with the glycoprotein spikes and the matrix layer beneath the viral membrane are clearly visible in the images. However, no major differences in the virions' morphology could be detected when comparing rDHOV(wt) with rDHOV(M2stop) and rDHOV(ΔM2) (Fig. 5b and c). Based on this limited data set (*n* = 40 for each recombinant virus), an axis ratio analysis suggests a marginally more elongated median axis ratio for rDHOV(ΔM2) when compared with rDHOV(wt) and rDHOV(M2stop). However, this difference was not significant (Fig. 5d).

In summary, our data show that the newly described M2-248 viral protein can interact with the viral M-270 protein and is a structural component present in extracellular virions. However, despite sharing a large, 185 aa long portion of identical sequence with M-270, M2-248 does not undertake the function of a viral matrix protein. It neither affected viral polymerase activity, assembly, or budding of virions nor the morphology of extracellular particles.

### rDHOV(ΔM2) is attenuated *in vivo*

**Fig 6:**
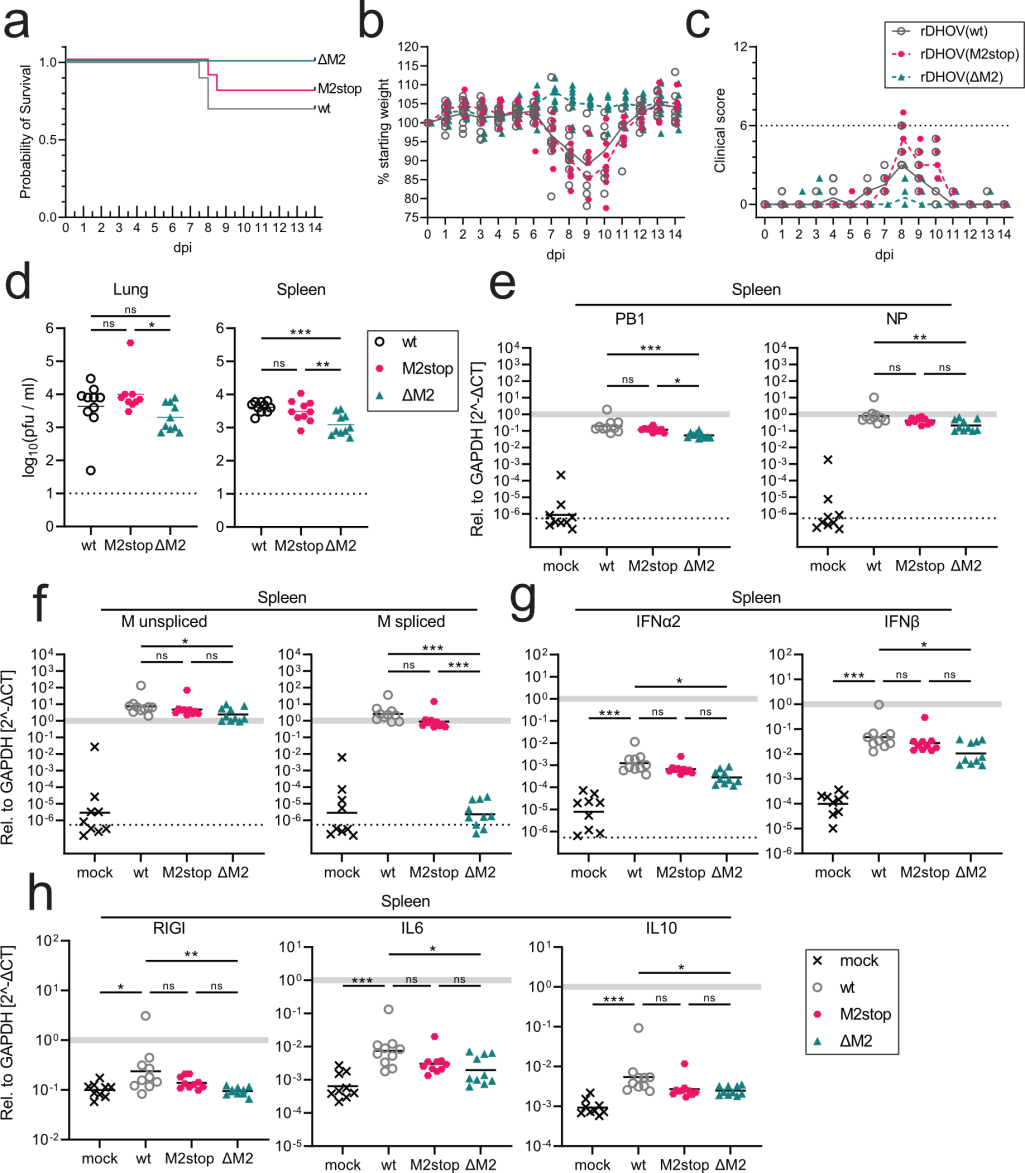
Virulence of rDHOVs *in vivo*. (a-c) C57BL/6 mice (*n* = 10 per group) were infected with 40 pfu of rDHOV(wt) (gray, circles), rDHOV(M2stop) (red, hexagons), and rDHOV(ΔM2) (turquoise, triangles) in 100 µL PBS via the intraperitoneal (i.p.) route. Survival (a), weight (individual and mean values) (b), and clinical score (individual and median values) (c) were monitored. Mice that showed severe clinical signs, according to clinical scoring parameters (see Fig.S3), sustained weight loss of >20% compared with their starting weight for more than 2 days or weight loss of >25% were euthanized. The dotted line in panel c indicates the clinical score at which mice had to be euthanized. (d) Replication of rDHOVs in lung and spleen. C57BL/6 mice (*n* = 9-10) were infected i.p. with 1,000 pfu of rDHOV(wt), rDHOV(M2stop), and rDHOV(deltaM2). At 4 dpi, the mice were euthanized. Lung and spleen were harvested and homogenized in PBS or in RNA-extraction buffer, respectively. Viral titers in the lung and spleen were determined using plaque assay and are displayed as log-transformed values for the individual animals with means ± SD. Statistical analyses were performed with a one-way ANOVA on log-transformed values [Tukey’s multiple-comparison test: *P* > 0.05 (ns), *P* ≤ 0.05 (*), *P* ≤ 0.01 (**), *P* ≤ 0.001 (***)]. (e, f) RT-qPCR analysis of viral RNA isolated from the spleen of infected mice (same animals as in panel d) using specific primer pairs amplifying RNA transcripts of the viral segment 2 (PB1), segment 5 (NP) (e), or segment 6 (f) either containing intron 2 (M unspliced) or missing intron 2 (M spliced). (g, h) Host transcripts of mouse IFNα2, IFNβ1, and RigI as well as pro-inflammatory IL6 and anti-inflammatory IL10 genes from the spleens. (e-h) Log 2^-Δct^ values were calculated relative to mGAPDH and displayed as log-transformed values on a linear scale. The gray bar indicates mGAPDH reference, and the dotted lines correspond to the detection limit (log_10_(2^-(CT_40_-avg.CT)_mGAPDH_)). Data points below the dotted lines indicate no amplification. Statistical analysis: ordinary one-way ANOVA [Tukey’s multiple-comparison test; *P* > 0.05 (ns), *P* ≤ 0.05 (*), *P* ≤ 0.01 (**), *P* ≤ 0.001 (***)].

To determine the impact of M2 expression on the virulence of rDHOV, we used laboratory mice as an *in vivo* model. Previous experiments with DHOV/India/1313/62 showed high virulence of this virus strain upon intraperitoneal (i.p.) infection with high virus loads in the liver, lung, and spleen and a fatal outcome within 5–6 days post-infection (dpi) (6, 14). Therefore, we infected C57BL/6 mice i.p. with a dose of 40 pfu of the rDHOVs (Fig. 6a through c) and determined the body weight and clinical score (Fig. S3j) daily, over 14 days. In case of severe disease symptoms, the animals were euthanized. Upon infection with rDHOV(wt) and rDHOV(M2stop), the animals developed severe symptoms including a decline in their body weight starting at 7–8 dpi when three and two of the 10 infected animals had to be euthanized, respectively (Fig. 6a through c). In parallel, animals were infected with 4 or 400 pfu (Fig. S3a to c, g to i). Infections with the reduced dose of rDHOV(wt) and DHOV(M2stop) resulted in the transient manifestation of disease symptoms, but only two out of 10 animals had to be euthanized around 8 dpi. However, infections with a high dose of 400 pfu resulted in higher mortality: six out of 10 infected animals for rDHOV(wt) as well as rDHOV(M2stop) succumbed to the infection. Using these results, we calculated a mouse lethal dose 50 (LD_50_) for rDHOV(wt) of ~186 pfu and for rDHOV(M2stop) of ~241 pfu. In contrast, the rDHOV(ΔM2)-infected animals showed only mild clinical symptoms around 8 dpi, and all animals survived the infection even with the high dose of 400 pfu (Fig. 6a through c and Fig. S3a through i), indicating an LD_50_ for rDHOV(ΔM2) far above 400 pfu.

In a second set of *in vivo* experiments, C57BL/6 mice were infected i.p. with a higher dose of 1,000 pfu but only for 4 days, to determine virus replication in different organs. As described before (6, 14), we detected a systemic infection of the animals with rDHOV(wt), detecting progeny virus and viral RNA in the lung, spleen, and liver (Fig. 6d and e and Fig. S4). Infection with rDHOV(M2stop) resulted in comparable titers like wild-type in the lung, spleen, and liver. However, for rDHOV(ΔM2), we detected slightly reduced viral replication (Fig. 6d and e). Of note, intron 2-spliced transcripts were not detected in rDHOV(ΔM2)-infected animals by RT-qPCR (Fig. 6f). Viral replication was not detected in the kidney and brain of the infected animals (data not shown).

Li and colleagues reported the elevated expression of inflammatory cytokines during experimental infection of mice with a lethal dose of DHOV/India/1313/62 that reflects the fulminant, systemic disease of the animals (15). Therefore, we also monitored cytokine induction in the organs of the rDHOV-infected animals at 4 dpi by RT-qPCR analysis of the RNAs isolated from the spleen, lung, and liver. rDHOV(wt) and rDHOV(M2stop) infections induced a clear upregulation of proinflammatory cytokine expression including IFNα2, IFNβ, and IL6 in the spleens of infected animals when compared with the mock-treated animals (Fig. 6g and h). These expression levels were only slightly reduced in the organs of rDHOV(ΔM2)-infected animals (Fig. 6g and h). Interestingly, the induction of RIGI, as a type I IFN-induced gene, and of IL-10 in the spleen remained almost unchanged. Analyses of RNAs extracted from the infected lungs and livers showed only low levels of cytokine induction and no differences between the three rDHOVs (Fig. S4a and b), most likely reflecting reduced levels of viral replication in these organs. Overall, the small differences in viral replication and cytokine induction between the three rDHOVs do not reflect the strong differences in inducing severe disease in the infected animals.

**Fig 7:**
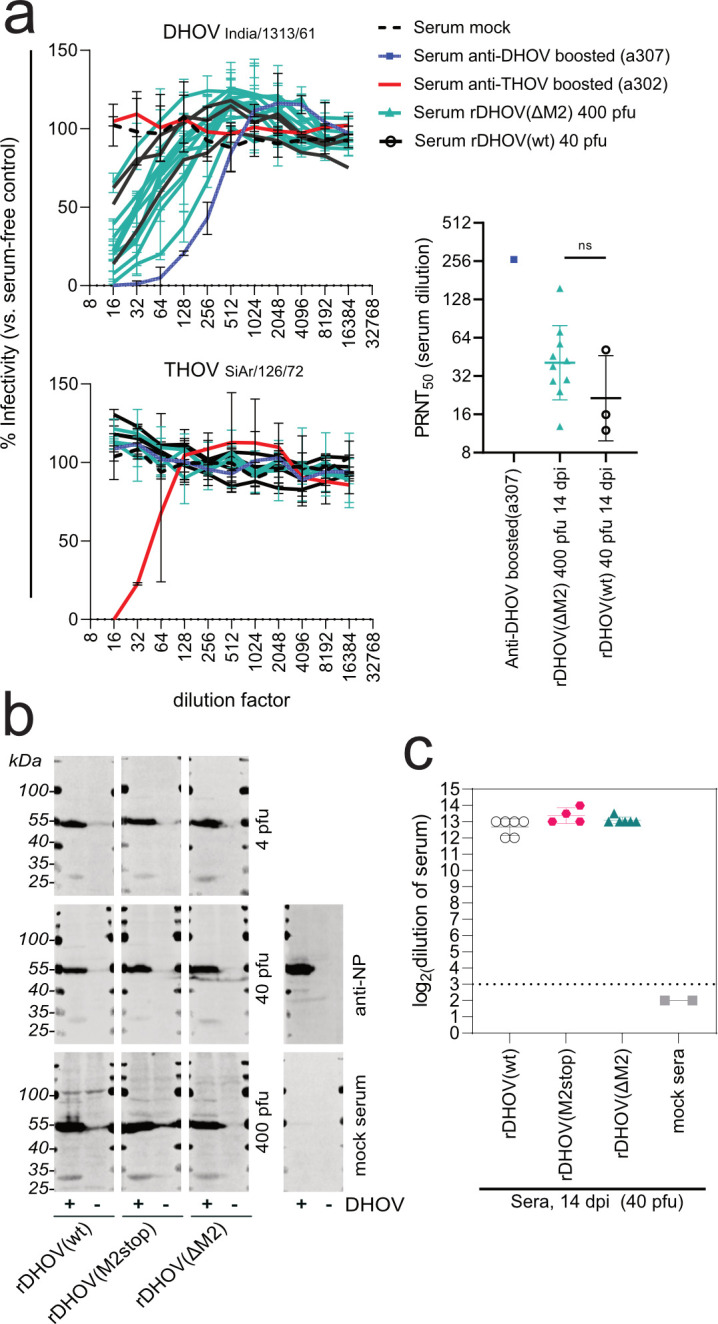
Characterization of sera from rDHOV reconvalescent mice. At 14 dpi, whole blood was obtained from mice by cardiac puncture from which serum was prepared. (a) Serial 2-fold dilutions of postinfectious rDHOV sera or control sera (mock or previously characterized anti-DHOV/THOV sera [6]) were incubated with PBS containing ~100 pfu of DHOV/India/1313/61 (upper panel) or THOV/SiAr/126/72 (lower panel) and analyzed by plaque assay (two replicate/serum). Relative infectivity was determined compared with serum-free control wells. The resulting PRNT_50_ (right panel, geometric mean ± geometric SD) was calculated from a four-parameter logistic regression. (b) Sera were analyzed for the recognition of viral proteins by western blot: A549 cells were infected with India/1313/61 at moi 5 or mock infected for 20 h. Four sera of each cohort were combined for the staining (serum dilution 1:1,000 each). A polyclonal DHOV-NP-specific rabbit serum (anti-NP) and a mock-infected serum were included as controls. (c) Titers of IgG recognizing viral antigens were determined by incubation of PFA fixed and permeabilized DHOV-infected Huh7 cells (moi 5, 20 hpi) with serial 2-fold dilutions of the postinfectious sera, followed by the detection using fluorescent-labeled anti-mouse IgG secondary antibodies. The sera of mock-infected animals were included as a negative control. Displayed are the highest dilutions at which a fluorescence signal was still detected for the individual serum (log2-transformed geometric means of technical duplicates for each animal as well as mean ± SD for each group).

To confirm the successful infection of the rDHOV(ΔM2)-inoculated animals, we harvested sera from the surviving animals at 14 dpi and tested for neutralization and general seroconversion. Plaque reduction neutralization assays showed robust neutralization of DHOV infectivity by the reconvalescent sera of rDHOV(wt) and rDHOV(ΔM2)-infected mice, with mean plaque reduction neutralization titers (PRNT_50_) of 1:40 or 1:28, respectively (Fig. 7a, upper and right panels). Expectedly, neutralization was specific for DHOV and did not impact THOV infectivity, as quantified for a subset of sera (Fig. 7a, lower panel). DHOV-specific IgG was found in the sera of all surviving animals infected with the three viruses at each dose, confirming successful infection. IgG in the sera predominantly recognized bands corresponding in size to NP (~52 kDa) and M-270 or M2-248 (~28-30 kDa) proteins in the western blot analysis (Fig. 7b). Finally, an immunofluorescence assay of DHOV India/1313/61-infected cells showed detection of viral antigens at high dilutions (1:4,096 to 1:16,384) of the post-infectious sera (Fig. 7c).

### Lack of IFN-antagonistic activity of DHOV M2-248

**Fig 8:**
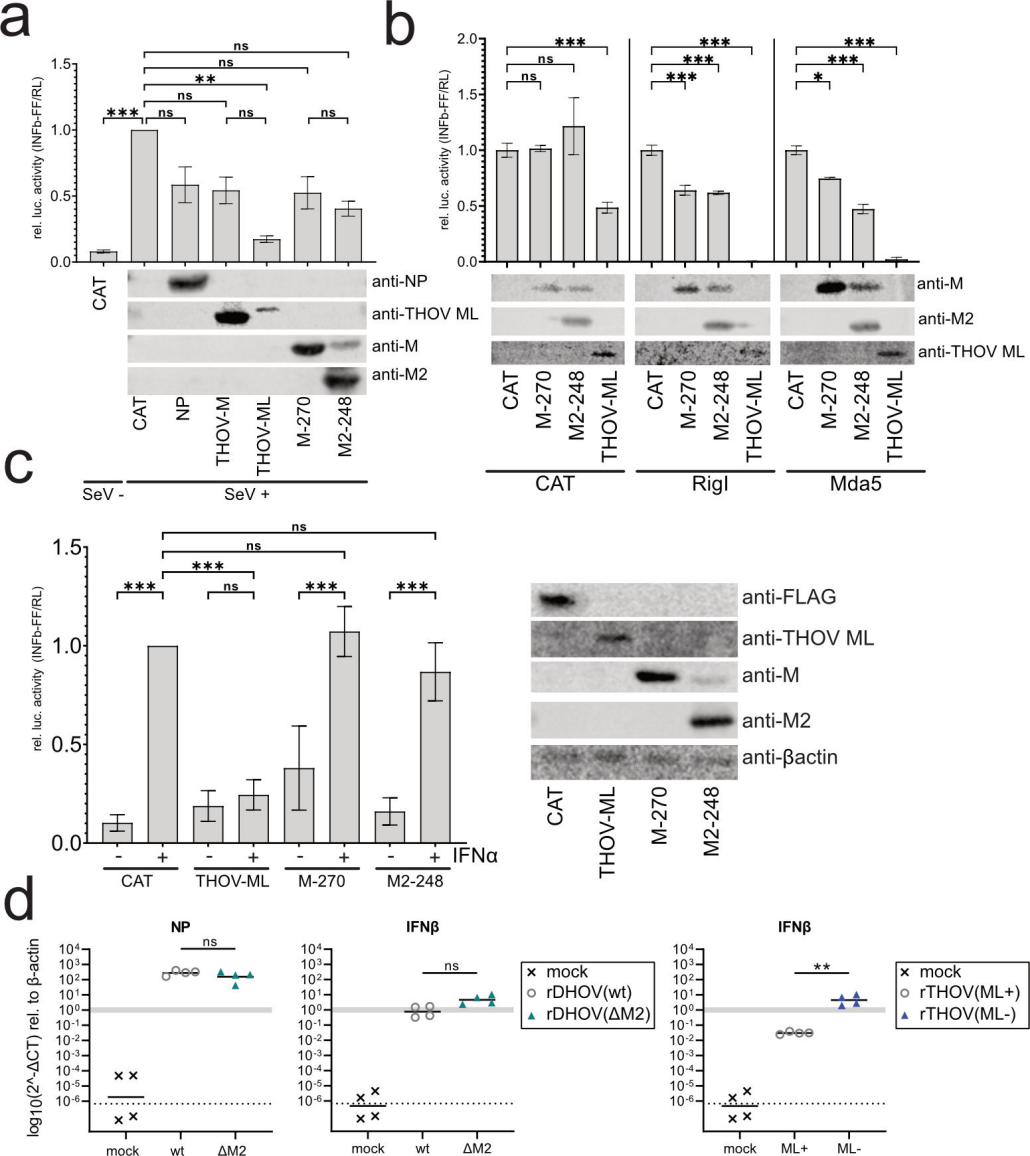
Investigation of IFN-antagonistic activity of DHOV M-270 and M2-248. (a, b) Effect of M-270 and M2-248 on IFN induction. 293T cells were transfected with an IFNβ-promoter firefly luciferase (FF) reporter construct (IFNβp-FF), a constitutive *Renilla* luciferase (RL) construct (SV40p-RL) as well as expression constructs for M-270 and M2-248, and chloramphenicol acetyltransferase (CAT) or DHOV-NP as negative controls, respectively. In parallel, the cells were transfected with expression constructs for THOV-M and THOV-ML, the interferon antagonist of THOV (17). (a) At 6 h post-transfection, the cells were infected with Sendai virus (SeV, moi 1.0) to activate the IFN promoter. Cells were lysed at 20 hpi, and luciferase activities were measured. (b) The effect of M-270 and M2-248 on IFN induction by RIG-I and MDA5. 293T cells were transfected as in (a). The IFN promoter was activated by co-transfection of RIGI-N or MDA5 expression constructs, respectively. Cells were lysed at 24 h post-transfection. FF luciferase values were normalized to *Renilla* luciferase (FF/RL) and then to the FF/RL of the CAT control average for each inductor (relative luc. activity). (c) The effect of M-270 and M2-248 on IFNα signaling. 293T cells were transfected with an *Mx1*-promoter FF luciferase reporter construct (Mx1p-FF), SV40p-RL, as well as the expression constructs for CAT, M-270, M2-248, or THOV-ML, respectively. At 24 h post-transfection, the cells were treated with IFNα2a (10 ng/mL) for 24 h before measuring luciferase activities. FF luciferase activities were normalized to RL luciferase (FF/RL) and to the FF/RL normalized mean of the IFNα2-treated CAT control (relative luc. activity). (a-c) Shown are the results of biological triplicates with technical duplicates. Expression of DHOV-NP, M-270, M2-248, and THOV-M/ML was validated using western blot analysis. (d) RT-qPCR analysis of transcripts in A549 cells infected with rDHOV(wt), rDHOV(ΔM2), or rTHOV-ML+ and rTHOV-ML- (17) with MOI of 1.0 for 24 h. RNA was isolated from the cells, and RT-qPCR was performed with primers specific to detect viral transcripts of the NP segment and cellular *IFNβ*. Log 2^-Δct^ values were calculated relative to the housekeeping gene, β-actin, and displayed as log-transformed values on a linear scale. The gray bar indicates the housekeeping gene reference, and the dotted lines correspond to the detection limit (log_10_(2^-(CT_40_-avg.CT_housekeeping_)). Data points below the dotted lines indicate no amplification. Statistical analysis: ordinary one-way ANOVA [Tukey’s multiple-comparison test; *P* > 0.05 (ns), *P* ≤ 0.05 (*), *P* ≤ 0.01 (**), *P* ≤ 0.001 (***)].

Like DHOV M2-248, THOV encodes an additional gene product on the viral segment 6. This THOV ML protein was characterized as a potent type I IFN antagonist (17, 19), and recombinant rTHOV-ML-, lacking this antagonistic activity, was found to be attenuated *in vivo* (31). Therefore, we hypothesized that the M2-248 gene product encoded by DHOV might also interfere with type I IFN induction or signaling. To get an idea of the potential IFN-antagonistic activity of DHOV M2-248, IFNβ promoter activation was determined in a luciferase reporter assay. To this end, 293T cells were co-transfected with a plasmid coding for firefly luciferase under the control of the human IFNβ promoter and expression constructs for DHOV NP, M-270, and M2-248 as well as the THOV M or ML protein. To activate the IFNβ promoter, the cells were infected with Sendai virus (SeV) or co-transfected with a constitutively active N-terminal fragment of RIG-I or MDA5, two sensors of intracellular viral RNA, leading to type I IFN induction. Upon stimulation, the IFNβ promoter was highly activated, resulting in elevated expression of firefly luciferase in the cells transfected with a CAT plasmid as an internal control (Fig. 8a and b). Co-transfection of DHOV NP reduced IFNβ promoter activation by 40%–50%, whereas THOV ML showed a strong effect as reported previously (17). However, the expression of DHOV M2-248 reduced the reporter gene expression by only 40%–50% and was not specifically different in comparison to the co-expressed M-270 or NP (Fig. 8a and b). Furthermore, we evaluated the effect of M2-248 on type I IFN signaling by using an IFN-sensitive, murine *Mx1* promoter reporter construct. Upon treatment with IFNα, the reporter expression was stimulated in the presence of the CAT control and was suppressed by co-expressed THOV ML protein (Fig. 8c). However, the increase of reporter activity upon IFNα treatment was not reduced by co-expressed M2-248 or the M-270 control (Fig. 8c).

To evaluate the effect of M2-248 on IFN induction in infected cells, A549 cells were infected with the recombinant DHOV either expressing M2-248, that is, rDHOV(wt), or lacking M2-248 expression, that is, rDHOV(ΔM2) for 24 h. Detection of viral NP expression confirmed comparable replication of the two viruses (Fig. 8d). rDHOV(wt) infection led to the upregulation of IFNβ transcripts, as reported previously (32). Infection with rDHOV(ΔM2) did only slightly increase endogenous IFNβ expression (Fig. 8d). As a control, we used recombinant THOV either expressing or lacking ML (17). As expected, A549 cells infected with rTHOV(ML-) showed elevated induction of IFNβ expression when compared with rTHOV(wt) infection (Fig. 8d). In summary, M2-248 lacks a significant effect on IFN induction or signaling in transfected or infected cell cultures matching the lack of *in vivo* effects of M2-248 deletion on the cytokine induction in rDHOV-infected animals (Fig. 6g and h; Fig. S4).

## DISCUSSION

Most animal viruses encode accessory proteins that support intracellular viral replication or act as viral antagonists of the innate or adaptive host defense (33). An example of such an accessory viral protein within the Thogotovirus genus is the ML protein of the Thogoto-like virus clade. THOV and also Jos virus were shown to express the viral M protein from spliced transcripts of segment 6, whereas unprocessed transcripts code for an elongated M protein called ML (17, 18). In-depth characterization of the M and ML gene products of THOV revealed the matrix protein function of the M protein for virus assembly and budding and the IFN-antagonistic function of the ML protein (19, 34). However, Dhori-like thogotoviruses seemed to possess a fundamentally different coding strategy for segment 6 in that the M protein is expressed from the collinear, unprocessed transcripts of segment 6, and splicing of these transcripts had not been reported (1). In detail, Clay and Fuller reported the expression of a 270 aa long M protein encoded by the unprocessed transcripts of the prototype strain DHOV/India/1313/61 (20). In the present study, we show that the DHOV M protein functions as an orthomyxoviral matrix protein in that it is found as a prevalent, structural component of purified extracellular particles of DHOV-infected cells and supports the viral budding process when co-expressed with the viral glycoprotein in a cell culture-based VLP system. Thereby, the DHOV M protein functions like the M protein of THOV and the M1 protein of IAV. Despite only low sequence similarities between 15% and 25% with the matrix proteins of IAV and THOV, a structural prediction of the DHOV M protein shows a two-domain structure very similar to the other orthomyxoviral M proteins (25, 27).

Clay and Fuller already described an additional ORF encoded by segment 6, overlapping with the M-ORF and spanning 141 amino acids in the −1 frame (20). However, in their study, neither subgenomic transcripts of segment 6 nor a second gene product could be detected in DHOV-infected cells. The results of the present study show that transcripts of DHOV segment 6 are spliced in a distinct double-intron pattern, that is, to our knowledge, unique among orthomyxoviruses, in a process that occurs in mammalian as well as tick cells. Furthermore, we demonstrate that splicing of intron 2 on segment 6 transcripts causes the shift into the −1 frame, leading to the synthesis of a novel viral protein, M2-248. The presence of this protein, consisting of the unique M2 region fused to the N-terminal M protein moiety, was confirmed in a range of infected mammalian cells. A putative gene product of double-spliced transcripts (M2-93) was not observed despite the relative abundance of this mRNA species on the amplicon level. A polypeptide of this size might be rapidly degraded in infected cells and thus not detectable in western blot analyses.

Our newly described splicing of segment 6 explains why Clay and Fuller failed to detect the expression of an M2-containing protein because the antigenic peptide sequence used to produce M2-specific antibodies (20) was localized within the intron 2 sequence that is absent in M2-248. A search of public databases for sequences related to the unique M2 region of M2-248 revealed no significant amino acid homology to other viral or cellular polypeptides. However, the M2 sequence is quite conserved among members of the Dhori-like clade, arguing for a significant role in DHOV replication.

Purification of DHOV particles and co-sedimentation of the virion structural proteins revealed that M2 is a structural component of extracellular particles and can interact with the M protein. However, our VLP assay system showed that M2-248 alone or in combination with M-270 was not able to support the budding of infectious particles, although M2-248 has the first 187 amino acids in common with the M-270 protein and shows a similar 3D fold in its N-terminal part. This is reminiscent of the ML protein of THOV that consists of the complete viral M protein fused to additional, C-terminal 38 amino acids, unique for ML (17). Similarly, ML was found in viral particles together with M but did not support virus budding in a VLP assay (34). Furthermore, the growth of a recombinant DHOV lacking M2-248, rDHOV(ΔM2), was not attenuated in cell culture, and virion morphology was not altered when compared with rDHOV(wt). Overall, this argues against a crucial function of M2-248 for viral assembly, contrasting the function of the M2 protein of IAV, which assembles a membrane-spanning homotetrameric ion channel critical for virus entry and release (35). Accordingly, M2-248 does not show any sequence similarity to the IAV M2 protein, and the AlphaFold prediction of the M2-248 structure did not show a hydrophobic pattern that could serve as a transmembrane domain.

The function of M2-248 could also be discussed in its property as an M-270-like protein, which is missing some of the properties of the canonical matrix protein, such as the putative viral late domains that were predicted for the M-270 C-terminal region for the recruitment of components of the ESCRT-complex. Thereby, M2-248 could serve as a “decoy” matrix protein that shares some functions with M-270 but does not partake in the recruitment of the ESCRT-complex, thereby fine-tuning the budding process.

The strong attenuation of the rDHOV(ΔM2) virulence *in vivo* was unexpected because viral polymerase reconstitution assays, VLP assays analyzing the production of infectious viral particles, and growth kinetics of M2-defective rDHOV(ΔM2) in cell culture revealed that M2-248, and canonical splicing of DHOV segment 6, is without a significant influence on viral replication. Furthermore, infection of mice with rDHOV(ΔM2) revealed only slight differences in tissue tropism and viral replication in different organs when compared with rDHOV(wt)-infected animals. Interestingly, the virulence of rDHOV(M2stop), a variant encoding a C-terminally truncated M2 due to an artificial, premature stop codon in the M2-ORF, without affecting splicing of segment 6, was unaltered in virulence when compared with rDHOV(wt). This strong virulence of DHOV(M2stop) suggests that either the truncated M2 sequence is sufficient for the function of M2 or that the intron 2 splicing event itself is crucial for DHOV to cause severe disease symptoms.

It has been shown that infection with DHOV causes a systemic spread of the virus and a severe fulminant fatal illness in mice (6, 13, 14) with strong cytokine induction, resembling a “cytokine storm” known from infections with highly pathogenic IAVs (15). Therefore, we expected the reason for the attenuated virulence of rDHOV(ΔM2) to be due to a reduced induction of inflammatory cytokines. However, analysis of cytokine expression in different organs of the infected animals did not indicate obvious differences that could account for the differences in the course of the infections. Additionally, we detected no clear effect of M2-248 expression on IFN induction or IFN signaling in transfected cells. Only in A549 cells did we detect slightly increased levels of IFNβ induction by rDHOV(ΔM2) infection; however, these differences were not significant and not matching the effect observed for THOV ML-.

In the light of the recent emergence of zoonotic Dhori-like thogotoviruses, such as Bourbon virus (BRBV) in the USA and Oz virus in Japan, *in vivo*-attenuated viruses analogous to rDHOV(ΔM2) might serve as a blueprint for the development of live-attenuated vaccine candidates since the mutant virus replicated readily in cell culture and evoked a robust production of neutralizing antibodies in mice.

In summary, we identified a new gene product of DHOVs, called M2-248, that is encoded by a spliced transcript of segment 6. The splicing process causes a shift of the M-ORF into a −1 frame coding for a new C-terminal appendix to the truncated M sequence.

Despite animal experiments indicating a role of M2 in DHOV virulence *in vivo*, our assay systems did not provide conclusive evidence for the function of this newly identified gene product. However, given the evolutionary pressure to conserve only functional proteins, especially within DHOV’s compact genome and considering the presence of a sophisticated splicing mechanism, future studies will likely uncover the biological significance of M2-248 for DHOV replication and for the switch between mammalian and tick hosts.

## MATERIALS AND METHODS

### Biosafety

All work with thogotoviruses was performed under biosafety level (BSL) 2 conditions, except for the human isolate of BRBV that was handled under BSL3 conditions. Generation of recombinant rDHOV and the manipulation of segment 6 of the viral genome were approved by the authorities of Baden-Wuertemberg, Germany (Regierungspraesidium Tuebingen, permit UNI.FRK.05.22-89/05.16-26/05.23-21).

### Cell lines, tick cells, and IFN treatment

Human lung epithelial A549 cells (ATCC CCL-185), human embryonic kidney HEK-293T cells (ATCC CRL-3216), human hepatoma Huh7 cells (36), Syrian golden hamster kidney cells BHK-21 (ATCC CCL-10), and African green monkey kidney Vero cells (ATCC CCL-81) were cultivated in Dulbecco’s Modified Eagle Medium (DMEM, Gibco; 41966-029) supplemented with 5%–10% fetal calf serum (FCS) and antibiotics (100 units/mL of penicillin and 100 µg/mL of streptomycin) at 37 ˚C and 5% CO_2_. 293T cells were treated with recombinant human IFN-α2a (PBL assay science).

Tick cell cultures. HAE/CTVM9 from *Hyalomma anatolicum anatolicum* (37) and RAE/CTVM1 from *Rhipicephalus appendiculatus* (38) (kindly provided by L. Bell-Sakyi, Tick cell biobank, University of Liverpool, UK) were propagated either in L-15/MEM or L15 medium, respectively, each supplemented with 20% FCS and 10% Tryptose phosphate broth. Cells were grown at 33°C (HAE) and 28°C (RAE) without additional CO_2_ as described previously (38). For infection, about 10^5^ cells were seeded in flat-sided tubes. The medium was almost completely removed, and the virus inoculum of 10^4^ pfu was supplied in a small volume of used medium for 2 h. Then, the infection medium was replaced by a mixture of conditioned and fresh medium. At 8 dpi, the cells and culture supernatants were collected and used for RNA isolation.

### Viruses

For the present study, we used DHOV/India/1313/61, kindly provided by Fred J. Fuller (7), Batken virus (BTKV, strain LEIV306K), kindly provided by Robert E. Shope (39), and PoTi461, kindly provided by Armindo R. Filipe (40), as well as the Dhori-like Bourbon virus (BRBV strain Kansas, NR-50132, ATCC VR-1842), kindly provided by Amy J. Lambert and Brandy Russell (11), and Oz virus (OzV, isolate number 264.1), kindly provided by Kyoko Sawabe (21). Sendai virus (SeV) strain Cantell (41) was used for IFN induction experiments. Virus stocks were produced on Vero or BHK-21 cells, and viral titers were determined by plaque assay on Vero cells as described previously (6).

For virus infection experiments including growth kinetics, the cells were seeded to 90% confluency overnight in 6-well cavities and infected with the respective virus diluted in 500 µL OptiMEM (Gibco, 11058-021) for 2 h at 37°C and 5% CO_2_. Afterward, the cells were washed three times with PBS and incubated with DMEM containing 2% FCS, 20 mM HEPES, and 0,1% NaHCO_3_. Viral titers were determined by plaque assay on Vero cells as described previously (6).

Inactivation of DHOV was performed by UV irradiation (two times 900 µJ/cm^2^; Ultra.LUM, UVC-515 Multilinker) of infectious cell supernatants on ice. Virus inactivation was confirmed by direct plaque assay of the treated supernatants or by incubation of BHK-21 cell cultures with the treated material for 6 days followed by plaque assay on Vero cells.

### Purification of extracellular DHOV virions

BHK-21 cells were infected with a moi of 0.001 of DHOV/India/1313/61 for 48 h. The rDHOV virions were propagated on Huh7 cells for 60 h. The cell supernatants were collected and centrifuged at 2,000 × *g* for 30 min at 8°C. Then, the supernatants were subjected to ultracentrifugation through a 30% glycerol cushion in PBS at 100,000 × *g* (SW32, Beckman Coulter) for 90 min at 8°C. The pellets were resuspended in a small volume of PBS and subjected to a discontinuous 30%–60% sucrose gradient in PBS with centrifugation (SW41) at 100,000 × *g* for 90 min at 8°C. The virion-containing band at the interface between 30 and 40% sucrose was aspirated, diluted with PBS, and sedimented through a 30% glycerol cushion in PBS (TLA55) at 100,000 × *g* for 40 min at 8°C. The resulting pellet was resuspended in a small volume of PBS and analyzed using SDS-PAGE and western blot.

### Virion lysis and glycerol gradient centrifugation

The resuspended pellet from the TLA55 centrifugation, about 3 × 10^7^ pfu in 500 µL, was mixed 1:1 with 2× lysis buffer: 100 mM Tris (7.5); 200 mM NaCl; 10% glycerol; 10 mM MgCl_2_; 2 mM DTT; 1% NP40; 2% Triton X-100; 20 mg/mL Lysolecithin (Sigma-Aldrich L5254); and protease inhibitor cocktail (cOmplete, Merck, Darmstadt, Germany) and incubated for 20 min at 30°C. Then, the suspension was cleared by centrifugation at 5,000 × *g* for 20 min at 8°C. The supernatant was applied on top of a discontinuous 33%–73% glycerol gradient: 1 mL steps of 33%, 43%, 52%, and 73% glycerol in 50 mM Tris (7.5), 150 mM NaCl, 1 mM MgCl_2_, and 1 mM DT and centrifuged at 135,000 × *g* (SW55) for 5 h at 8°C. Then, the tube was dropped out in 10 fractions of 500 µL, with fraction number one at the bottom (73%) and fraction number 10 at the top (5%). The single fractions of the gradient were analyzed by SDS-PAGE and western blot using DHOV-NP-, M-, and M2-specific antisera and a monoclonal anti-β-tubulin antibody as a marker of cellular protein contaminations.

### Cloning of viral cDNAs and expression of recombinant proteins

To clone the cDNAs of the viral genome segments, Vero cells were infected with DHOV/India/1313/61 (moi of 1) for 24 h. RNA was isolated using the NucleoSpin RNA Kit (Macherey-Nagel, 740955.50) according to the manufacturer’s protocol. Total RNA (1 µg) was reverse transcribed using the QuantiTect Reverse Transcription kit (Qiagen). The ORFs of DHOV PB1, PB2, PA, GP, NP, and M were amplified by PCR using KOD hot start polymerase (Sigma-Aldrich) and specific primer pairs (Table S1). For cloning the different splice variants of segment 6, the amplicons were separated by agarose gel electrophoresis, and the bands were purified according to their different sizes using the Zymoclean™ Gel DNA Recovery Kit (Zymo Research). The isolated amplicons were digested and ligated into the digested and dephosphorylated pCAGGS expression vector (42) via T4 DNA ligase (Thermo Fisher Scientific). The sequences of all cloned cDNAs were confirmed by Sanger sequencing.

For cloning of the rescue plasmids of DHOV/India/1313/61, the cDNAs encoding the six genomic segments were amplified using primers complementary to the individual non-coding regions of the segments (Table S1) and cloned into ambisense pHW2000 vector, kindly provided by R. G. Webster (30).

For site-directed *in vitro* mutagenesis, a two-step PCR was performed, in which the target gene was first amplified in two parts with internal primers harboring the desired nucleotide substitutions. These two parts of the insert were used as templates in a subsequent second PCR to generate the full-length target sequences. The inactivating mutations of the splice donor and acceptor sites of segment 6 were introduced using specific primer pairs (Table S1): Splice acceptor site of intron 1 (genomic nt position A516C; primer pair 2724/2725), the splice donor site of intron 2 (genomic nt position C591A + G594A; primer pair 2718/2719), and splice acceptor site of intron 2 (genomic nt position G750A; primer pair 2720/2721), yielding pHW-Seg6(ΔM2). A premature stop codon was introduced into the M2-ORF at genomic position CAC846-848TAG (corresponding to the aa change in M2-248 of E218*) of the viral cDNA to yield pHW-Seg6(M2stop), using primer pair 2716/2717. For the corresponding protein expression vectors, the mutated ORFs were amplified and cloned into the pCAGGS vector.

For the expression of recombinant viral proteins, adherent, nearly confluent 293T cells were transfected with plasmid DNA and jetPEI (Polyplus) transfection reagent according to the manufacturer’s instructions. For western blot analysis, the cells were lysed at 24–48 h post-transfection, as indicated.

### Generation of recombinant DHOV

rDHOV(wt), rDHOV(M2stop), and rDHOV(ΔM2) were generated as described previously for rTHOV (19). The pHW2000 plasmids encoding PB1-, PB2-, PA-, GP-, and NP-segments (1–5) and the respective wild-type or mutated M-segment (segment 6) of DHOV/India/1313/61 were transfected (500 ng/each) (Lipofectamine 2000, Thermo Fisher Scientific) into a 2:1 co-culture of 293T and Vero cells (~1 × 10^6^ cells per 6-well). At 72 h post-transfection, the supernatant was harvested, and rDHOV was purified by plaque assay on Vero cells. Each recombinant virus was independently rescued twice. Virus stocks of rDHOV were produced by transferring single plaques to fresh BHK-21 cell cultures. The sequence of segment 6 in progeny viruses of the second cell culture passage and the presence of the introduced mutations were verified by RT-PCR and sequencing of the cDNAs.

### Viral polymerase reconstitution system and virus-like particles (VLPs)

To reconstitute the polymerase activity of DHOV/India/1313/61, 293T cells (~4 × 10^5^ cells per 12-well) were co-transfected (JetPEI; Polyplus) with 10 ng of pCAGGS expression plasmids encoding the polymerase subunits PB2, PB1, PA, and 50 ng of NP plasmids as previously described for THOV (43). In addition, 50 ng of an artificial viral minigenome encoding firefly luciferase (FF) in negative-sense orientation flanked by the 5’- and 3′-NTRs from DHOV segment 5 (pPolI-FF) and 10 ng of a plasmid coding for a *Renilla* luciferase (RL) under the constitutive SV40 promoter (SV40p-RL) were added. At 24 h post-transfection, FF and RL luciferase activities were measured (Dual-luciferase reporter kit; Promega). FF luciferase activity was normalized to RL luciferase activity (FF/RL).

For the production of replication-incompetent virus-like particles (VLPs), 293T cells (~5 × 10^5^ cells per 6-well) were transfected with pCAGGS expression plasmids encoding the structural proteins of DHOV/India/1313/61: 20 ng of PB2, PB1, PA, 100 ng of NP, 75 ng of pPolI-FF for the vRNA minigenome, and 5 ng of SV40p-RL as a transfection control. In addition to the components of the polymerase reconstitution system, expression plasmids encoding the viral glycoprotein, GP (50 ng), and the viral matrix proteins, M-270 (50 ng), and M2-248 (100 ng), were co-transfected. At 48 h post-transfection, 293T cell culture supernatants were harvested and cleared by centrifugation at 2,000 × *g* for 20 min at 4°C. The expression of the viral proteins in the transfected cell cultures was monitored using western blot analysis of the lysed 293T cells using NP-, M-, and M2-specific antibodies and anti-β-actin as a loading control. Formation of infectious VLPs was determined by transferring the cleared supernatants onto BHK-21 indicator cells. VLP formation was monitored by the detection of FF luciferase activity in the BHK-21 cells 48 h post-transfer.

### Immunoblotting and antibodies

Infected cells were lysed with a 1:1 mix of T-PER tissue protein extraction reagent and SDS sample buffer (Thermo Fisher). Cell lysates of the polymerase reconstitution assays were mixed with a 2-fold SDS sample buffer. Following full denaturation at 95°C for 5 min, the samples were separated by SDS polyacrylamide gel electrophoresis (SDS-PAGE, 10% acrylamide). The separated proteins were transferred onto a PVDF membrane (Merck). The membranes were first blocked with blocking buffer (0.1% Tween-20, 5% milk powder in PBS) for 1 h and then stained with the primary antibody for 1 h at RT, followed by incubation with secondary, fluorescent-labeled antibodies (LI-COR) for 1 h at RT as well. The antibodies were diluted in blocking buffer, and in between the staining steps, the membranes were washed three times for 10 min with washing buffer (0.1% Tween-20 in PBS). In the case of the anti-M2 staining for western blot analyses of viral particles (Fig. 4a and b), an HRP-coupled, secondary antibody (Agilent/Dako, ref. P0448) was used for higher sensitivity. Finally, the membranes were washed 4 times for 5 min with a washing buffer, and fluorescent or chemiluminescence signals were detected using the LI-COR Odyssey Imaging System (LI-COR, Lincoln, NE, USA).

Primary antibodies used were as follows: anti-SiAr126 NP (rabbit, polyclonal [44]), anti-JOSV (mouse, polyclonal [6]), anti-FLAG M2 (mouse, monoclonal, Sigma-Aldrich), anti-β-actin (rabbit, polyclonal, Abcam), and anti-β-tubulin (mouse, monoclonal, Sigma-Aldrich). Anti-DHOV NP antiserum (rabbit, polyclonal) was raised against the purified His-tagged NP produced in *Escherichia coli* as described previously (44). Rabbit, polyclonal antiserum detecting THOV M and ML was raised against the purified His-tagged M protein produced in *E. coli* (45). To generate DHOV anti-M antiserum, the rabbits were immunized with purified viral matrix protein, isolated from extracellular viral particles. Viral proteins of lysed particles were subjected to SDS-PAGE, and upon Coomassie staining, the M protein band was cut out from the gel, resuspended in PBS, and used for immunization of rabbits (Davids Biotechnologie, Regensburg, Germany). A polyclonal anti-M2 specific rabbit serum was raised by immunization with an LPH-conjugated peptide corresponding to M2-248(194-207) (BioGenes, Berlin, Germany). The specificity of the DHOV-specific anti-NP, -M, and -M2 antisera was confirmed using western blot analysis of lysates from transfected cells expressing the recombinant viral proteins.

### Fluorescence microscopy

For immunofluorescence microscopy analysis, Huh7 cells were seeded onto coverslips and infected. The cells were fixed in paraformaldehyde (4% in PBS) for 15 min at RT and washed with PBS. Afterwards, the cells were permeabilized with Triton X-100 (0.5% in PBS) and washed again with PBS. After blocking for 1 h with blocking buffer (PBS with 1% BSA and 0,1% Tween 20), the coverslips were incubated with the primary antibody for 1 h at RT, followed by washing five times with PBS. The secondary fluorescence-labeled antibody was incubated in the dark at RT for 1 h. After washing once with PBS, the cells were stained with DAPI (4’,6-diamidino-2-phenylindole) (0.3 mM in PBS) for 10 min, washed again three times with PBS, and mounted onto microscope slides using FluorSave (Millipore). Pictures were taken with an LSM 880 AiryScan (Carl Zeiss, Jena, Germany). Polyclonal rabbit antisera directed against DHOV NP, M, and M2 were preadsorbed to naive, fixed and permeabilized Huh7 cells for 24 h at 8°C prior to using them as primary antibodies for immunofluorescence.

For the investigation of seroconversion in reconvalescent mice, confluent Vero cells in 96-well plates were infected with DHOV/India/1313/61 at moi 5. After 20 h, the cells were fixed with 4% PFA, and staining was conducted as above with 2-fold serial dilutions of the respective mouse sera and an Alexa Fluor 488-coupled donkey-anti-mouse-IgG secondary antibody (A21202; Invitrogen).

### Scanning electron microscopy (SEM)

Huh7 cells were grown to 60% confluency on 18 × 18 mm indium tin oxide-coated coverslips (SPI supplies, #06465-AB, 8-12 Ω) and treated with BHK-21 mock-supernatant or rDHOVs at a MOI of 4. At 24 hpi, the cells were washed once with PBS and fixed with 4% paraformaldehyde (PFA) and 0.5% glutaraldehyde (GA) in PBS for 1 h at RT. Coverslips were then processed for scanning electron microscopy (SEM). Coverslips were washed with 0.1 M Cacodylate buffer. Next, the cells were incubated with 1% OsO4 at 4°C for 30 min, followed by washing with Cacodylate buffer. Dehydration was done in acetone solutions with increasing concentrations of acetone (25%, 50%, 75%, 95%, and 100%) and incubation for 10 min. Critical point drying was done on a Leica CPD300 at 17°C and 63.5 bar followed by sputter coating with a 5 nm thick layer of Au/Pd (80/20) using the Leica ACE600. Samples were mapped by SEM using an Aquilos 2 dual-beam cryo-focused ion beam-scanning electron microscope (ThermoFisher Scientific) operated at room temperature at magnifications between 10,000× and 25,000× and five keV, using OptiPlan with a working distance of 3 mm and in-column T2 secondary electron detector. SEM images were acquired in the MAPS software (ThermoFisher Scientific).

### Cryo-electron tomography (cryo-ET)

BHK-21 cells were infected with the respective viruses at moi 0.001 and incubated for 72 h with DMEM supplemented with 2% FCS, 20 mM HEPES, and 0.1% NaHCO_3_. Supernatants containing >1 × 10^6^ pfu/mL of infectious particles were UV-inactivated (two times 900 µJ/cm^2^) on ice. Successful inactivation was confirmed by plaque assay on Vero cells. The virus-containing supernatant was mixed with 10 nm protein A-coated colloidal gold (Aurion). The mixture (3–4 µL) was applied onto 200 mesh, copper R2/1 grids (Quantifoil), which were plasma cleaned using H_2_/O_2_ mix for 10 seconds using Solarus plasma cleaner (Gatan). Plunge-freezing into liquid ethane was performed using an automatic EM GP2 plunge-freezing device (Leica) under the following conditions: chamber temperature: 25°C, humidity: 80%, back-side blotting: 3 s. Grids were stored in liquid nitrogen until cryo-transmission electron microscopy.

Cryo-ET data were collected using a Titan Krios transmission electron microscope (ThermoFisher Scientific) operated at 300 keV and equipped with a Quanta Imaging Filter (Gatan) with an energy filter slit set to 20 eV and a K3 direct electron detector (Gatan). Grids were mapped at 8,700× magnification (pixel spacing: 10.64 Å) to localize virions, and tilt series were acquired at 33,000× magnification (pixel spacing: 2.67 Å) in SerialEM (46) using a dose-symmetric tilting scheme (47), nominal tilt range from 60° to −60° and 3° increments, target defocus −3 µm, electron dose per record 3 e^-^/Å^2^. Tomograms were reconstructed in Etomo using weighted back projection with simultaneous iterative reconstruction technique (SIRT)-like filter equivalent to seven iterations, dose-weighting, and 2D contrast transfer function (CTF) correction. The length and diameter of virions from the outer membrane to the outer membrane were measured in IMOD (48).

### IFN induction and signaling reporter assays

To measure the influence of DHOV M-270 and M2-248 on IFN induction, 293T cells (~3 × 10^4^ cells per 96-well cavity) were co-transfected with the following plasmids using FuGeneHD (Promega): 10 ng of p125-Luc (IFNβp-FF) encoding firefly luciferase (FF) under the control of the IFNβ promoter (49) and 4 ng of SV40p-RL, constitutive expression of *Renilla* luciferase (RL)(Promega), as well as 20 ng of each pCAGGS expression plasmid encoding FLAG-tagged bacterial chloramphenicol acetyltransferase (CAT), as a negative control, DHOV-NP (pCAGGS-DHOV-NP), DHOV M-270, DHOV M2-248, HA-tagged THOV-M (pCAGGS-M), or THOV-ML (pCAGGS-M5xTΔSA [17]), as a positive control. At 6 h post-transfection, the cells were infected with moi 1.0 of Sendai virus (SeV) strain Cantell (19) to activate the IFNβ promoter. At 18 hpi, the cells were lysed, and FF as well as RL luciferase activities were measured (Dual-luciferase reporter kit; Promega). FF luciferase was normalized to RL luciferase activity (FF/RL) and is indicated as a relative activity of 1.0 for the CAT control. Expression of the recombinant DHOV-NP, M-270 and M2-248, and THOV-ML were validated by western blot analysis using specific antibodies. Detection of β-actin was used as a loading control.

In a second set of experiments, the IFNβ promoter was activated by co-transfection of 100 ng per 12-well cavity of expression plasmids pCAGGS-FLAG-RIG-I-N, encoding the constitutively active N-terminal domain of the intracellular RNA sensors RIG-I, or plasmid pEF-MDA5-Myc encoding Myc-tagged MDA5 (kindly provided by Rick Randall) (50). Expression of reporter gene activity was determined at 24 h post-transfection as described above.

To measure the effect of M-270 and M2-248 on IFNα signaling, the 293T cells were co-transfected with 10 ng per 96-well cavity of a reporter plasmid encoding FF luciferase under the control of the IFN-stimulated murine *Mx1* promoter, pGL3-Mx1p-FF (51), and 20 ng of the plasmids encoding the viral M proteins. At 24 h post-transfection, the cells were treated with 10 ng/mL of human IFN-α2a (PBL Assay Science), and activation of the *Mx1* promoter was determined by measuring luciferase activities in the cell lysates at 24 h post-treatment as described above.

### Co-immunoprecipitation

293T cells (~1 × 10^6^ cells per 6-well) were transfected with 1 µg of each pCAGGS expression construct coding for C-terminally FLAG- or HA-tagged M-270 and M2-248. As a negative control, chloramphenicol-acetyltransferase (CAT) N-terminally FLAG- or HA-tagged was used. At 48 h post-transfection, the cells were lysed for 10 min on ice in lysis buffer (50 mM Tris-HCl, pH 8.0, 20 mM NaCl, 0.2% NP-40, 1 mM DTT, and protease inhibitor cocktail [Merck, 11873580001]), adjusted to 150 mM NaCl and cleared by centrifugation for 10 min at 12,000 rpm at 4°C. Cleared supernatants were incubated with 13 µL of anti-FLAG-M2-affinity agarose beads (Sigma-Aldrich, A2220) for 3 h at 6°C under rotation. Whole cell lysates and FLAG-agarose precipitated proteins were denatured in SDS-sample buffer for 5 min at 95°C and analyzed by SDS-PAGE and western blot using DHOV-NP-, M-, and M2-specific antisera.

### Mass spectrometry (MS) analysis

To prove the expression of M2 on protein level in DHOV-infected cells, triplicates of A549 cells (~1 × 10^6^ cells per condition) were infected with DHOV/India/1313/61 at moi 1 or mock-treated. The cells were lysed in 0.5 mL of 1% SDS in PBS at 6 and 24 hpi. Lysates were separated by SDS-PAGE, and bands in the range of 0–35 kDa were excised from the gel and digested with trypsin. Resulting peptides were analyzed by LC-MS/MS on a Qexactive HF-X mass spectrometer coupled to an EasyLC 1200 nanoflow-HPLC (ThermoScientific). MS raw files were analyzed using MaxQuant (version 1.6.2.10) (52). MaxQuant results were analyzed using Perseus (v.1.5.5.3) (53) and Instant Clue (v.0.10.10.20210316) (54). iBAQ (intensity-based absolute quantification) values for DHOV M-270 and M2 as well as cellular GAPDH and β-actin were median normalized and log-transformed (55).

### RNA isolation and RT-PCR

RNA was isolated from cultured A549 cells or mouse organs using the NucleoSpin RNA mini kit (Macherey-Nagel). RNA concentration was determined using a NanoDrop photometer (Thermo Scientific).

Reverse transcription (RT) was performed using 10 ng of the purified RNA and the Qiagen RT-kit (Qiagen, Germany; cat. 205313) with random hexamer primers. Upon inactivation of the RT reaction, the cDNA was used for PCR analysis using KOD-Polymerase (Sigma-Aldrich, cat. 71085) and the following primer pairs (Table S1) according to the manufacturer’s instructions (tm 58°C): DHOV/M-segment primer pair 1553/1554; DHOV/NP-segment 3011/1952; and human β-actin 560/561. PCR products were analyzed in a 1.5% agarose gel electrophoresis and stained with ethidium bromide (1:10,000). Bands were excised from agarose gels and purified with a gel purification kit (Zymo; cat. D4002 according to the manufacturer’s instructions). Gel-purified PCR products were used for cloning or for Sanger sequencing (Microsynth, CH).

### Quantitative RT-PCR (RT-qPCR)

For the quantitative analysis of viral and host gene expression, qPCR was performed with the PowerUp SYBR Green master mix (Thermo Fisher Scientific, Waltham, MA, USA; Cat. A25918) on a Quant Studio 5 system (ThermoF.). For the detection of DHOV transcripts, the following primer pairs were used (see Table S1): DHOV segment 2 #3005/#3006; segment 5 #3011/#3012; and segment 6 (unspliced) #2587/#2590. To detect splicing of the second intron in transcripts of segment 6, a primer specific for the splice junction site #2586 was combined with the reverse primer #2590 specific for exon 3.

For the detection of cytokine induction, commercially available primer sets (Qiagen, Venlo, NL) were used for the quantification of host transcripts: Mm_Ifna2 (QT00253092), Mm_ifnb2 (QT00249662), Mm_Il6 (QT00098875), Mm_Il10 (QT00106169), Mm_Ddx58 (RIGI, QT00123515), Mm_Isg15 (QT00322749), and Hs_IFNB1 (QT00203763).

Ct values of technical replicates were averaged, and changes in gene expression were determined using the 2−ΔCt method relative to the expression of the indicated housekeeping genes: Mm_Gapdh_3 (QT01658692) or human β-actin.

### Next-generation sequence analysis (NGS)

For the sequencing of virus stocks and the supernatant of DHOV-infected tick cells, RNA was purified using the Quick-RNA Viral Kit (Zymo Research, Irvine, CA, USA; R1035) with DNase I treatment (Zymo.; E1010), followed by DNAse I treatment with RiboLock (Thermo Fisher Scientific, Waltham, MA, USA; ref. 89836). The NEBNext Ultra II Directional RNA Library Prep kit was used to prepare the library (NEB #E7760, E7765). After quality assessment of DNA in a Bioanalyzer 2100 DNA Chip (Agilent, Santa Clara, CA, USA), quantification, and standard normalization, sequencing was conducted in a MiSeq (Illumina, San Diego, CA, USA).

### Mouse infections

Age and sex-matched C57BL/6 mice (7–9 weeks old) were purchased from Janvier Labs (Mayenne, FR). The animals were infected intraperitoneally (i.p.) with DHOV diluted in 100 µL PBS or mock injected with 100 µL PBS. For the determination of the median lethal dose (LD50), weight and disease symptoms were monitored daily. If the weight loss was greater than 25% of the mice showed severe disease symptoms (ruffed fur, lethargy, and hunched posture; Fig. S3), they were killed by cervical dislocation. For organ titers, liver, lung, spleen, kidney, brain, and serum were harvested at day 4 post-infection and homogenized with 800 µL PBS in 2 mL screwcap tubes with ceramic beads in a FastPrep Homogenizer (MP Biomedicals, Irvina, CA, USA). After centrifugation at 5,000 rcf for 10 min at 4°C, virus titers in the supernatants were analyzed by plaque assay on Vero cells. For the preparation of RNA from the lung and spleen, organs were lysed in 600 µL RA1 buffer (Macherey Nagel, Düren, DE; ref. 740961) using the FastPrep Homogenizer and further processed according to the manufacturer’s instructions. For the preparation of RNA from the liver, organs were first homogenized in 1 mL TRItidy G^TM^ (AppliChem, Barcelona, ES). Homogenates were then mixed with 200 µL Chloroform, vortexed, and clarified at 12,500 rcf at 4°C for 15 min. The RNA-containing aqueous phase was then mixed 1:3 with RA1 buffer and processed as above. Sera of surviving animals were obtained at day 14 post-infection and analyzed for seroconversion to DHOV-specific IgG antibodies that recognize viral antigens in an immunofluorescence assay and a western blot analysis using DHOV-infected cell lysates (6).

### Plaque reduction assay

Neutralizing antibody titers in post-infectious (14 dpi) sera of mice infected with rDHOV(wt) (infectious dose 40 pfu) and rDHOV(ΔM2) (infectious dose 400 pfu) were determined against DHOV and THOV by a plaque reduction assay. Previously characterized anti-DHOV and anti-THOV mouse sera served as controls (6). Serial 2-fold dilutions of the sera in PBS were incubated for 1 h at room temperature with 100 pfu of DHOV/India/1313/61 and THOV/SiAr/126/72, respectively. The serum-virus mixture was used to infect Vero cells for 90 min at 37°C. The inoculum was removed, and the cells were overlaid with 0.6% Oxoid agar for 72 h at 37°C. Cells were fixed with 3.7% formaldehyde and stained with 0.1% crystal violet. The reduction in counted plaque numbers was determined in comparison to an infected, serum-free control. To evaluate the neutralizing capacity, the plaque reduction neutralizing titers 50 (PRNT_50_) were determined by performing a non-linear fit least squares regression (constraints: 0 and 100). Each serum was tested twice against both viruses.

### Bioinformatical methods

Protein structures for DHOV/India/1313/61 M-270 and M2-248 as well as THOV(SiAr126) M and ML were modeled using AlphaFold3 (https://golgi.sandbox.google.com) (24). Processing and annotation of protein structure graphics were handled in ChimeraX (V1.4 (56). Hydrophobicity plots according to Kyte & Doolittle (57) (https://web.expasy.org/cgi-bin/protscale/protscale.pl) and molecular weights (https://web.expasy.org/compute_pi/pi_tool-ref.html) were predicted using the Expasy Bioinformatics toolkit (58). Motifs and domains were predicted with InterProScan (https://www.ebi.ac.uk/interpro/result/InterProScan) (59), and alignments of protein sequences were generated with Clustal Omega (https://www.ebi.ac.uk/jdispatcher/msa/clustalo) within the EMBL-EBI Job Dispatcher sequence analysis tools framework (60).

### Statistical analyses

Data were visualized and statistically evaluated with GraphPad Prism 9.4.1. Statistical tests were performed as indicated in the figure legends.

## Data Availability

GenBank accession numbers are as follows: for DHOV/India/1313/61 cDNAs, MT628428 to MT628433; for Segment 6 sequences of BKNV, MT628421; for PoTi461, MT628427; for BRBV, MT628415; and for OzV, LC320128 (as described in reference 6).

## SUPPLEMENTAL MATERIAL

Supplemental material jvi.00600-25-s0001.pdf Figures S1 to S4; Table S1.

ASM does not own the copyrights to Supplemental Material that may be linked to, or accessed through, an article. The authors have granted ASM a non-exclusive, world-wide license to publish the Supplemental Material files. Please contact the corresponding author directly for reuse.
