# Supplementary material for: A novel accessory gene product of tick-borne Dhori-Orthomyxovirus, encoded by overlooked spliced transcripts of RNA segment 6
Source: J Virol. 2025 Sep 15;99(10):e00600-25. doi: 10.1128/jvi.00600-25 (PMC12548436; doi:10.1128/jvi.00600-25)
Supplement: Supplemental material — Figures S1 to S4; Table S1. [file jvi.00600-25-s0001.pdf]

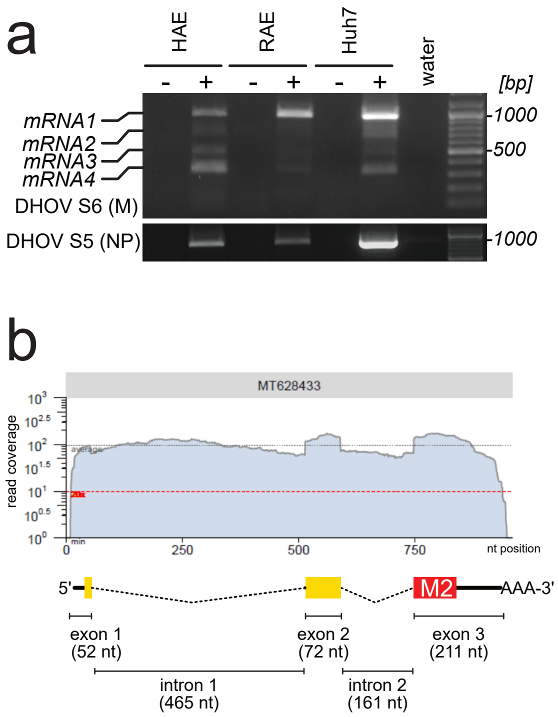

Figure S1: Segment 6 splicing pattern in tick cells. HAE/CTVM9 cells from *Hyalomma anatolicum* and RAE/CTVM1 cells from *Rhipicephalus appendiculatus* were cultivated to semiconfluency. **(a)** The cells ($1x10^5$ cells in flat-sided tubes) were inoculated with DHOV/ India/1313/61 at an moi of 0.1 or mock infected for 8 days. RNA was isolated for conventional RT-PCR to visualize splicing of viral transcripts using the segment 6 spanning primer set (compare Fig. 1b) and a segment 5 specific primer set. RNA from Huh7 cells (moi 1, 16 hpi) was used as a control. **(b)** Infected HAE cell RNA was additionally analyzed in a next generation Illumina sequence analysis (NGS) and displayed as the coverage of segment 6 (MT628433). The structure of segment 6 transcripts and their splicing pattern are indicated below.

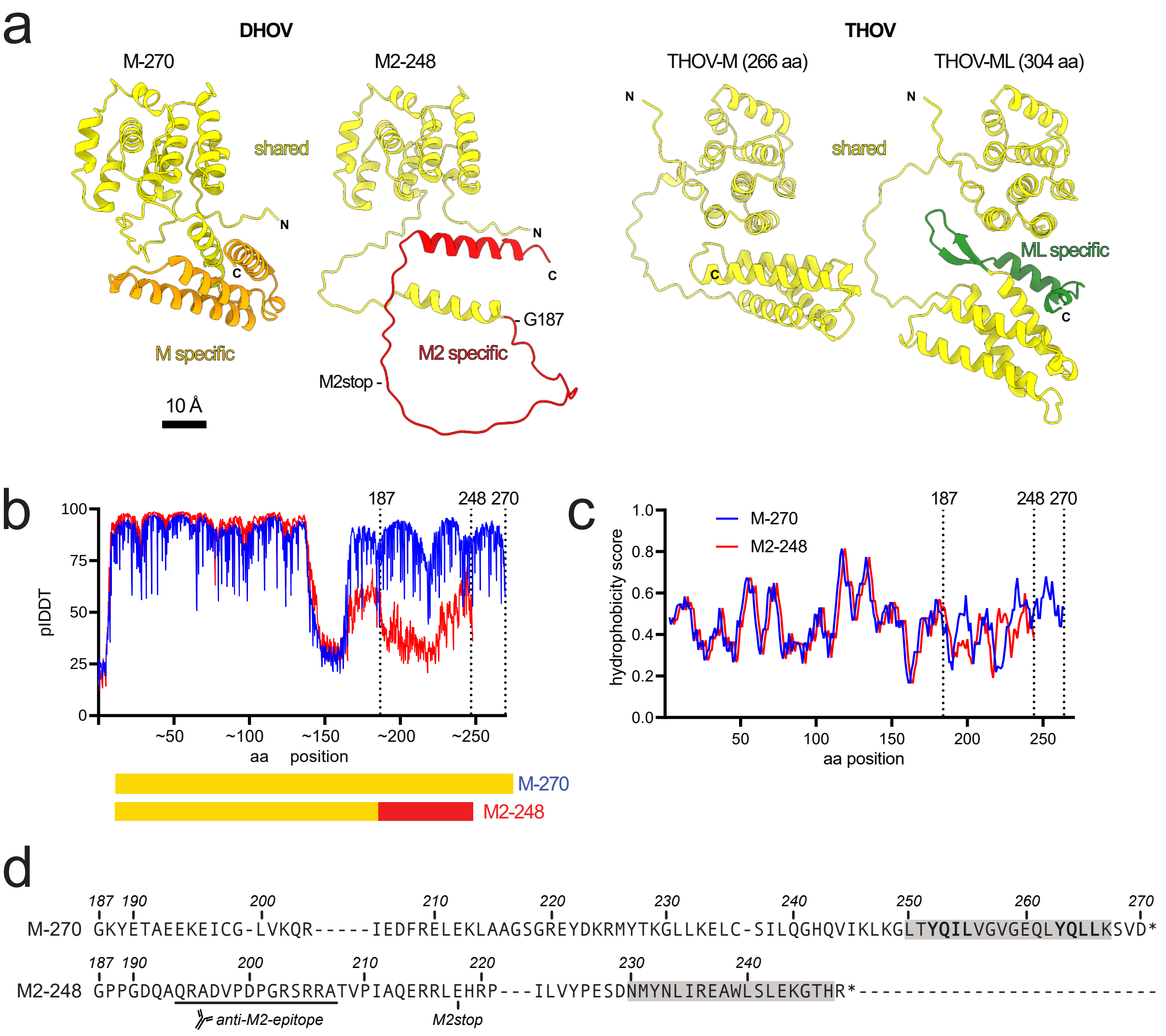

Figure S2: (a) *In-silico* structural analysis of matrix proteins encoded by the respective segments 6 of DHOV and THOV using AlphaFold 3 [1] and annotated in ChimeraX (V1.4). Protein structures of DHOV M-270 and M2-248 as well as THOV M (266 aa) and ML (304 aa). The N-terminal domains shared by the M proteins are indicated in yellow. The C-terminal domain of DHOV M-270 encoded by the unspliced intron 2 is indicated in orange. The C-terminal domain of M2-248 encoded by the unique M2-frame generated by intron 2 splicing is indicated in red. THOV M consists of 266 aa. THOV ML (304 aa) consists of M with a C-terminal extension of 38 aa indicated in green. **(b)** Predicted Local Distance Difference Test (pLDDT) score for the AlphaFold 3 structures of M-270 (blue) and M2-248 (red) shown in (a). A higher score indicates a higher confidence in the predicted structure. **(c)** Hydrophobicity score of M2-248 (red) compared to M-270 (blue) calculated according to Kyte&Doolittle [2] with a sliding window size of 9. A higher score indicates increased hydrophobicity. **(d)** Amino acid sequence alignment of the C-terminal M-270 and M2-248 specific regions. Amino acid positions are indicated above each sequence. Predicted amphipathic helices (grey boxes) and late-domain motifs (YxxL, underlined) are indicated. The alignment was generated with Clustal Omega MSA tool in the EMBL-EBI Job Dispatcher sequence analysis tools framework [3].

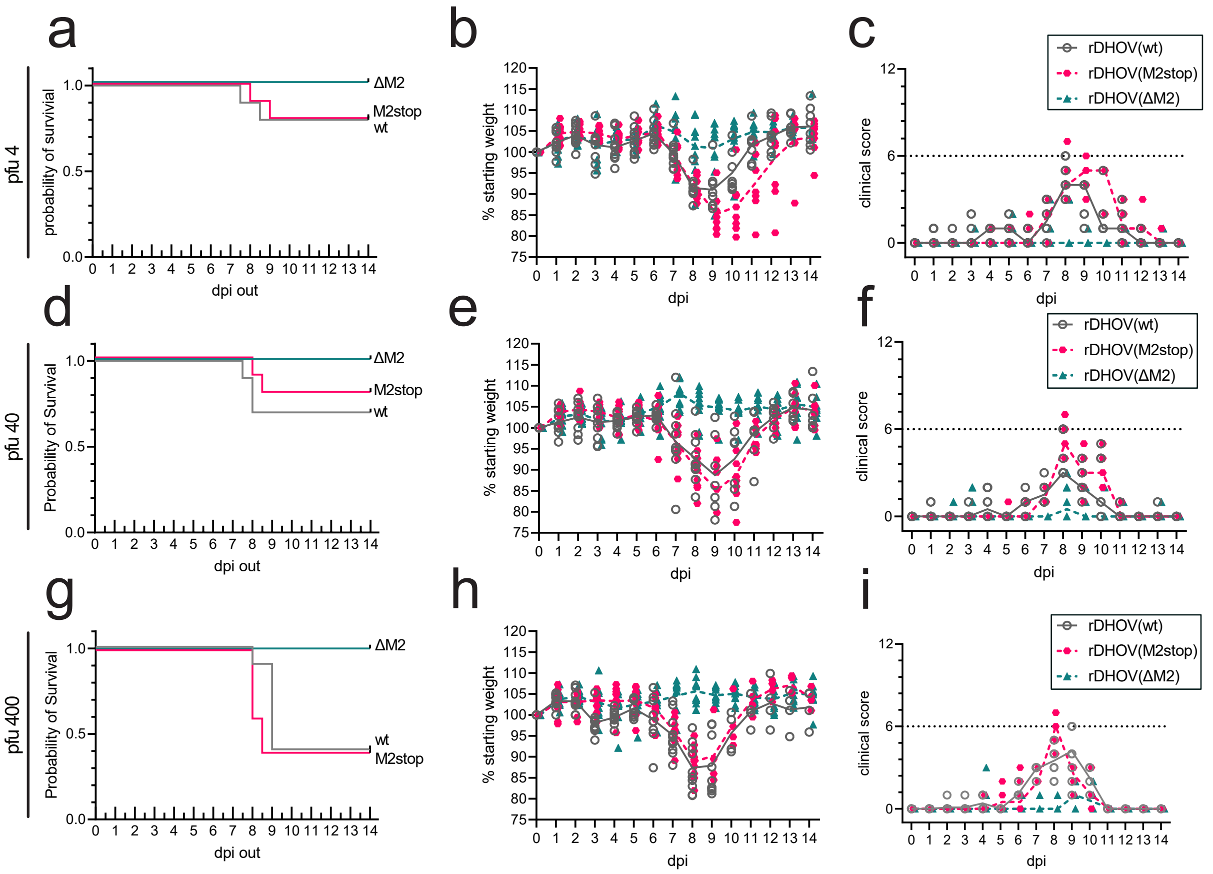

j

	Clinical scoring parameters	Score
Posture	normal<br>slightly hunched<br>hunched	0<br>1<br>2
Fur	smooth<br>patches of ruffed fur<br>completely ruffed fur	0<br>1<br>2
Eyes	open<br>slightly to moderately closed<br>completely closed with secretions	0<br>1<br>2
Spontaneous behavior	normal<br>reduced activity<br>no activity	0<br>1<br>2
Provoked behavior	immediate response to touch<br>moderate response to touch<br>little to no response to touch	0<br>1<br>2
Body weight loos	0 – 7.5%<br>7.5 -15%<br>>15%	0<br>1<br>2
	**Maximum score**	**12**

Figure S3: Virulence of rDHOVs. C57BL/6 mice (n = 10 per group) were infected with 4 pfu **(a-c)**, 40 pfu **(d-f)**, or 400 pfu **(g-i)** of rDHOV(wt), rDHOV(M2stop) and rDHOV(ΔM2) in 100 µl PBS via the i.p. route. For better comparison, data in panels d-f (40 pfu) were included from Fig. 4a-c. Survival (a, d, g), weight (individual and mean values) (b, e, h), and clinical scoring parameters (individual and mean values) (c, f, i) were monitored. Mice that showed severe clinical signs, according to clinical scoring parameters, sustained weight loss of >20% compared to their starting weight for more than 2 days or weight loss of >25% were euthanized. The dotted lines in panels c, f, i indicate the clinical score at which mice had to be euthanized. **(j)** Clinical scoring parameters (as described by [4]) are used to evaluate the critical disease state of the infected animals.

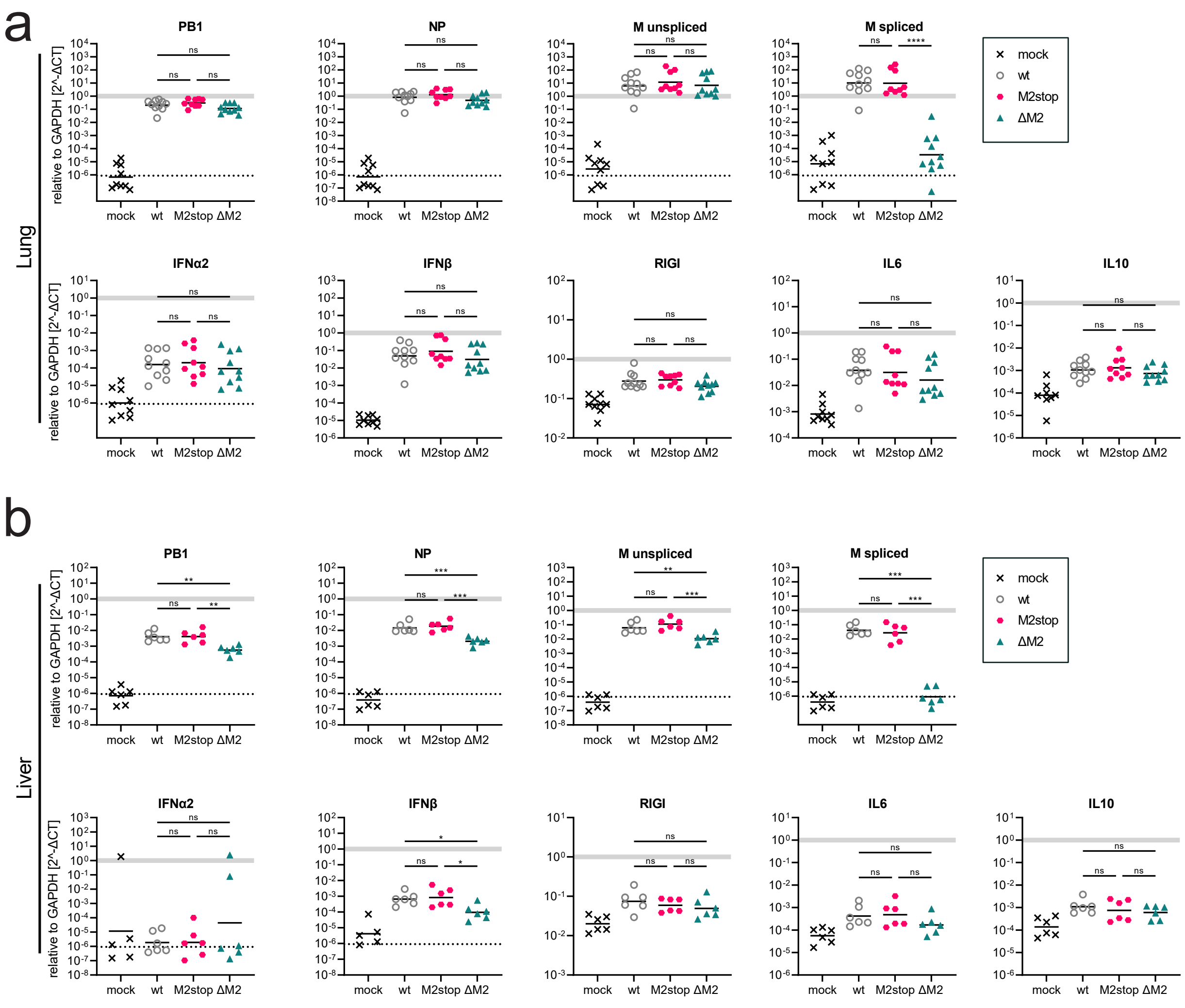

Figure S4: Organ tropism of rDHOVs. C57BL/6 mice (n = 9-10) were infected i.p. with 1000 pfu of rDHOV(wt), rDHOV(M2stop) and rDHOV(ΔM2) for 4 d. RT-qPCR analysis of viral and host RNA isolated from lung **(a)** and liver **(b)** of the same animals as in (Fig. 4d-h). Relative expression levels ($log2^{-\Delta ct}$) were calculated relative to mGAPDH and displayed as log-transformed values on a linear scale. The grey bar indicates mGAPDH reference and the dotted lines correspond to the detection limit ($log_{10}(2$^-$(CT_{40}$-avg.$CT_{mGAPDH}))$. Data points below this line indicate no amplification. Statistical analysis: Ordinary one-way ANOVA (Tukey's multiple-comparison test; $p > 0.05$ (ns), $p \leq 0.05$ (*), $p \leq 0.01$ (**), $p \leq 0.001$ (***)).

Table S1: Primers

Target	Forward primer (5´-3´)	Reverse primer (5´-3´)	Purpose
DHOV segment 6 (full)	AGCAATAACAAGCAGTACTAGACC	AGAGAAATCAAAGCAGTTTTTTCTCA	Analytic conventional PCR (splicing)
Human beta actin	GTTTTGGCGCTTTTGACTCAGGA	CTTTTGGGAGGGTGAGGGACTT	Analytic conventional PCR
DHOV NP-ORF	AGCTAAACTGCAAACCGGTG	GCCTCTCTGGGGTTTAGACCA	Analytic conventional PCR
DHOV Segment 6	CTCTGATTCCGAGGACGAG	CTCGTCCTCGGAATCAGAG	Mutating splice acceptor site of intron 1, generating Seg6(ΔM2)
DHOV Segment 6	CTAAAAGGAAAATACGAGACTGCG	CGCAGTCTCGTATTTTCCTTTTAG	Mutating splice donor site of intron 2, generating Seg6(ΔM2)
DHOV Segment 6	ATCCTGCAAGGCCACCAGG	CCTGGTGGCCTTGCAGGAT	Mutating splice acceptor site of intron 2, generating Seg6(ΔM2)
DHOV Segment 6	TCGATTAGAGTAGCGACCGATC	GATCGGTCGCTACTCTAATCGA	Premature M2 stop, generating Seg6(M2stop)
DHOV PB2-ORF	GACACCCGGGATGGATGTGCTGAAAGGAATTAAG	GACAGCTAGCCTAGTCTCTCTTTGTTGACGGTCC	Cloning into pCAGGS
DHOV PB1-ORF	GACACCCGGGATGAATCTCTTCTCTCCCAGAACAGG	GACAGCTAGCCTAACTATCCATCTCTTCCAGCAGAG	Cloning into pCAGGS
DHOV PA-ORF	GACACCCGGGATGGACCGACACAAGCCCAAGTC	GACAGCTAGCCTACATGTAGACATTTGGAAGC	Cloning into pCAGGS
DHOV NP-ORF	GACACCCGGGATGTCCTCGACAACTCCC	GACACTCGAGAGCTAAACTTCAAACAGAAGC	Cloning into pCAGGS (3373)
DHOV GP-ORF	GACACCCGGGATGGATTCCACCATCCGCCTAG	GACAGCTAGCTCAAAAAACTCTCCTCATTAGG	Cloning into pCAGGS
DHOV M-ORF	GATCGAATTCACCATGGCTGCCCACCAAATGGC	TCTCCTCGAGAGAAATCAAAGCA	Cloning into pCAGGS
DHOV Segment 1 (PB2)	GAGACGTCTCGGGGAGCAAAAACAAGCAGTTTAGACA	GAGACGTCTCGTATTAGAGAAATCAAAGCAGTTTTTCTTCAA	Cloning into pHW2000
	GGACATACTGTACTACACTGCA	GATCCTTTTTCTCGACGTATTGA	
DHOV Segment 2 (PB1)	GAGACGTCTCGGGGAGCAAAAACAAGCAGTTGACAGT	GTTCATCGAATTCTGAGGGCA	Cloning into pHW2000
	GGAGAAGGACTGACTTACCAGA	GAGACGTCTCGTATTAGAGATATCAAAGCAGTTTTTTCCTGA	
DHOV Segment 3 (PA)	GAGACGTCTCGGGGAGCAAAAACAAGCAGTTACCACT	CGGGAAGTTTGCACGAGTCCA	Cloning into pHW2000
	AGCGGAAGAGACAGGAAGAAGA	GAGACGTCTCGTATTAGAGAAATCAAAGCAGTTTTTTCCTA	
DHOV Segment 4 (GP)	GAGACACCTGCCATGGGGAGCAAAAACAAGCAGTTACGATGGA	GAGACACCTGCCATGTATTAGAGAAATCAAAGCAGTTTTTCTTCA	Cloning into pHW2000
DHOV Segment 5 (NP)	GAGACGTCTCGGGGAGCAATAACAAGCAGTTTCGAAAATGT	GAGACGTCTCGTATTAGAGATATCAAAGCAGTTTTTTAACA	Cloning into pHW2000
DHOV Segment 6 (M)	GAGACGTCTCGGGGAGCAATAACAAGCAGTACTAGACCA	GAGACGTCTCGTATTAGAGAAATCAAAGCAGTTTTTTCTCAA	Cloning into pHW2000
DHOV Segment 1 (PB1)	AGAAAGAGGACGGCTCAACA	AGGCACTCAAGCAATACCCT	qPCR
DHOV Segment 5 (NP)	AGCTAAACTGCAAACCGGTG	ATCCTCTGTCTTGACGCACA	qPCR
DHOV Segment 5 (NP)	~	GCCTCTCTGGGGTTTAGACCA	Conventional PCR
DHOV Segment 6 (M) unspliced	TGCAGCAGGTTCGGGGAGGGA	TCGGTCGGTGCTCTAATACGCT	qPCR
DHOV Segment 6 (M) spliced	CCCTCACACCCGTCCTAAAAGGGCC	~	qPCR

References

[1] J. Abramson *et al.*, “Accurate structure prediction of biomolecular interactions with AlphaFold 3,” *Nature*, vol. 630, no. 8016, pp. 493–500, Jun. 2024, doi: 10.1038/s41586-024-07487-w.

[2] J. Kyte and R. F. Doolittle, “A simple method for displaying the hydropathic character of a protein,” *J Mol Biol*, vol. 157, no. 1, pp. 105–32, May 1982, doi: 10.1016/0022-2836(82)90515-0.

[3] F. Madeira *et al.*, “The EMBL-EBI Job Dispatcher sequence analysis tools framework in 2024,” *Nucleic Acids Res*, vol. 52, no. W1, pp. W521–W525, Jul. 2024, doi: 10.1093/nar/gkae241.

[4] J. Fuchs, T. Straub, M. Seidl, and G. Kochs, “Essential Role of Interferon Response in Containing Human Pathogenic Bourbon Virus,” *Emerg. Infect. Dis.*, vol. 25, no. 7, pp. 1304–1313, Jul. 2019, doi: 10.3201/eid2507.181062.
